# Structural basis for tRNA mimicry by mascRNA and menRNA

**DOI:** 10.1038/s41421-024-00761-1

**Published:** 2025-01-02

**Authors:** Yuanlin He, Jie Deng, Xiaowei Lin, Zhizhong Lu, Liangliang Wang, Liang Xu, Yin Zhang, Jia Wang, Lin Huang

**Affiliations:** 1 Guangdong Provincial Key Laboratory of Malignant Tumor Epigenetics and Gene Regulation, Guangdong-Hong Kong Joint Laboratory for RNA Medicine, Sun Yat-Sen Memorial Hospital, Sun Yat-Sen University, Guangzhou, Guangdong China; 2 Department of Urology, Sun Yat-Sen Memorial Hospital, Sun Yat-Sen University, Guangzhou, Guangdong China; 3 Department of Urology, Dafeng Hospital, Chaoyang District, Shantou, Guangdong China; 4 School of Life Sciences and Biopharmaceutics, Guangdong Pharmaceutical University, Guangzhou, Guangdong China; 5 MOE Key Laboratory of Bioinorganic and Synthetic Chemistry, School of Chemistry, Sun Yat-Sen University, Guangzhou, Guangdong China; 6 Department of Cellular and Molecular Diagnostics Center, Sun Yat-Sen Memorial Hospital, Sun Yat-Sen University, Guangzhou, Guangdong China; 7 College of Pharmacy, Shenzhen Technology University, Shenzhen, Guangdong China

**Keywords:** X-ray crystallography, RNA editing

Dear Editor,

Within the human cell nucleus, two specific long noncoding RNAs, the metastasis-associated lung adenocarcinoma transcript 1 (MALAT1) and multiple endocrine neoplasia-β (MENβ), exhibit unique expression patterns linked to various cancers, metastasis, and poor prognosis^1–3^. Their maturation requires precise processing by RNase P. The MALAT1-associated small cytoplasmic RNA (mascRNA) and the MEN β-associated RNA (menRNA) are further processed by RNase Z and CCA addition, mimicking transfer RNA (tRNA) maturation (Fig. 1a)^4,5^.

**Fig. 1:**
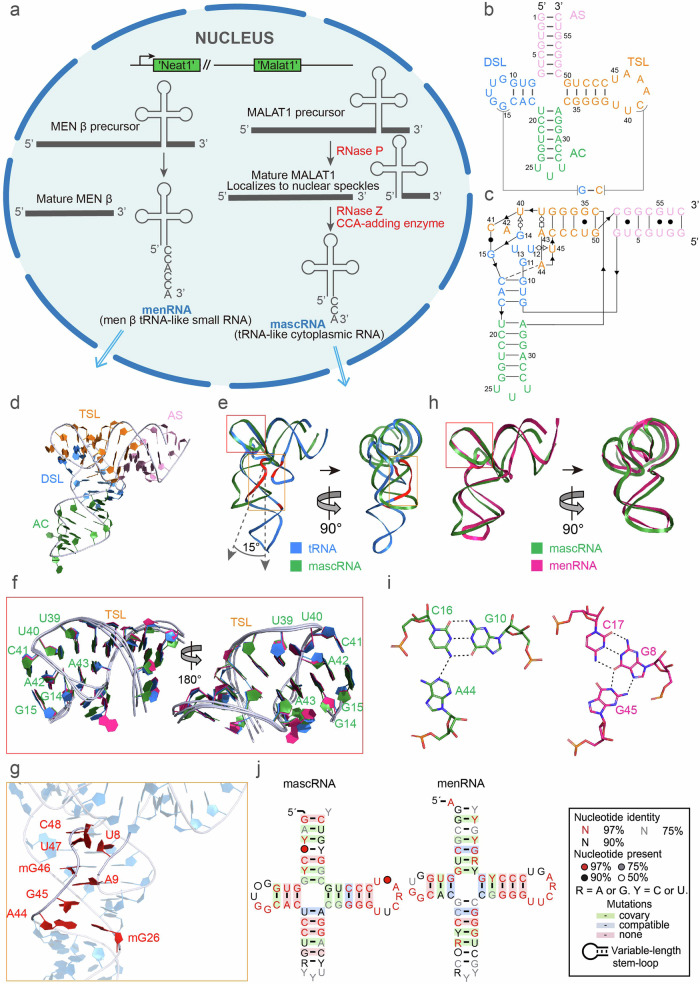
Comparison of human mascRNA with tRNA and menRNA. **a** Schematic representation of mascRNA and menRNA biogenesis. The MALAT1 precursor, transcribed from the *MALAT1* gene, undergoes processing by RNase P to simultaneously yield the mature MALAT1 and the 5’ end of mascRNA. Followed by RNase Z cleavage to generate the 3’ end of mascRNA and then CCA addition to the 3’ end of mascRNA to form mature mascRNA that is transported to the cytoplasm. The precursor of MEN β is processed through a pathway analogous to that of the MALAT1 precursor to generate menRNA. **b** The secondary structures of human mascRNA. (DSL: marine, AC: forest, TSL: orange, AS: pink). Long-range interactions between DSL and TSL are indicated. **c** Crystal structure-derived secondary structure of human mascRNA. Base-pairing symbols follow Leontis–Westhof symbols^11^. **d** Overall crystal structure of human mascRNA, colored to match the scheme used in **b**. **e**, **h** Two views of the structural overlay of human mascRNA (forest) and yeast tRNA^phe^ (marine) (**e**), human mascRNA and menRNA (hotpink) (**h**), superimposed on the TSL and two G. Red and orange boxes are shown in **f** and **g**, respectively. **f** Two views of structural superposition of the TSL and two G in human mascRNA, menRNA and yeast tRNA^phe^. **g** The eight tRNA linking residues absent in mascRNA are shown in red. **i** C16–G10–A44 base triplets and C17–G8–G45 base triplets at DSL and TSL in human mascRNA (left) and menRNA (right). **j** Consensus sequence and secondary structure model for the mascRNA and menRNA.

The secondary structures of mascRNA and menRNA are predicted to adopt a cloverleaf configuration, resembling that of canonical tRNA^3,4^ (Fig. 1b; Supplementary Fig. S1). This structure is hypothesized to fold into a three-dimensional conformation similar to the L-shaped arrangement of tRNA. However, the precise formation of a four-way junction from the secondary structure cannot be definitively deduced using only the sequence information. Even if such a junction is present, the three-dimensional structure may not necessarily conform to the L-shaped structural characteristic of tRNA.

Analyses of the secondary structures suggest that both mascRNA and menRNA have regions analogous to the acceptor stem (AS) and T stem-loop (TSL) of tRNA. However, the D stem-loop (DSL) regions are relatively smaller, and the anticodon stem (AC)-equivalent region features a longer stem and a compact loop. Notably, they lack the critical eight single-stranded linker residues found in tRNA’s four-way junction (Supplementary Fig. S1). These observations raise questions about the similarity of the tertiary structures of mascRNA and menRNA to that of tRNA.

We have determined the crystal structures of human mascRNA at 2.40 Å, human menRNA at 2.23 Å, and medaka mascRNA at 2.28 Å (Fig. 1c, d; Supplementary Tables S1, S2). To crystallize human mascRNA, we strategically mutated adenine to guanine at the second position (A2G) and removed the UCCA sequence from the 3′ terminus. This approach was instrumental in achieving the desired crystalline structure. The structure of medaka mascRNA at 2.28 Å was solved using the single-wavelength anomalous dispersion method (Supplementary Fig. S2), while the remaining structures were solved by molecular replacement, using the former as a reference model.

Both mascRNA and menRNA fold into a 3D L-shaped conformation characteristic of classical tRNA, featuring all tRNA equivalent regions. Specifically, for mascRNA, these regions span nucleotides 1–7 and 51–57 for AS, 34–50 for TSL, 8–18 for DSL, and 19–33 for AC; for menRNA, they are 1–7 and 52–58 for AS, 35–51 for TSL, 8–17 for DSL, and 18–34 for AC (Fig. 1b; Supplementary Fig. S1). The AS and TSL stack to form one continuous helical stack containing four GU base pairs, while the AC and DSL form another. The two helices are held in a roughly perpendicular orientation by interactions between the elements that are analogous to the D and T loops in tRNA (Fig. 1c, d; Supplementary Fig. S1). MascRNA and menRNA share a similar topological structure with tRNA, but the intramolecular interactions to form the structure are different from those in tRNA (Fig. 1c; Supplementary Fig. S1).

In our study, we conducted pairwise root-mean-square deviation (RMSD) analysis to explore the similarity between the structures of mascRNA, menRNA, and tRNA (Supplementary Fig. S3). Initially, our overall superposition revealed significant differences in the structures of these molecules. However, we observed a high degree of overall structural similarity between mascRNA and menRNA (Supplementary Fig. S3a). By gradually narrowing the superposition regions, we found that the structural variances between mascRNA, menRNA, and tRNA primarily reside in the AC. Additionally, we found that two conservative guanines in the D loop are essential for maintaining the tRNA-like structure (Supplementary Fig. S3b, c). Notably, our analysis revealed that the TSL + GG regions exhibit high similarity, with RMSD values within 3.0 (Supplementary Fig. S3d). Finally, with the TSL + GG as the basis, we achieved a more favorable superposition for all the structures (Supplementary Fig. S3f).

Compared to the tRNA structure, the TSL of mascRNA almost perfectly overlaps, whereas the DSL of mascRNA is shorter, with its lower half deviating by ~15° (Fig. 1e). Furthermore, the T loop of mascRNA exhibits a key interaction between nucleotides U39–A43 and two guanines in the D loop, echoing the tRNA’s elbow region. U39 and U40, the leading nucleotides of the T loop, are integrated into the stacking configuration of the T stem, and the nucleotide C41 pairs with G15 in the DSL through Watson–Crick base pairing. G14 is interposed between A42 and A43, creating a continuous stacking pattern that bridges the gap between these two nucleotides (Fig. 1f).

To validate the interactions in the elbow region that are crucial for mascRNA, we performed native polyacrylamide gel electrophoresis (Supplementary Fig. S4). We introduced mutations in the elbow region of mascRNA, including G15C, C41U, G14C/G15C, and UUCG loop substitutions of the D loop and T loop, respectively. The replacement of the D loop and T loop significantly accelerated migration, indicating that the disruption of key interactions at the elbow leads to changes in the overall folding of the mascRNA. The C41U mutation had a minor impact on the conformational change, likely because the replacement with a pyrimidine still permits the formation of a GU pair. In contrast, the G15C and G14C mutations, which disrupt elbow base pairing and continuous purine stacking, had a slightly greater impact on the conformation.

In the L-shaped structure of tRNA, the elbow region is stabilized by interactions between the V loop and the D stem (Fig. 1g). However, mascRNA lacks many of these key stabilizing interactions. Instead, it has a distinctive triple base interaction that provides structural integrity. This interaction involves a hydrogen bond from the N4 of C16 to the N1 of A44, along with a base pair formed between G10 and C16 (Fig. 1i). This alternative stabilization mechanism underscores the distinct structural characteristics of mascRNA.

MascRNA and menRNA share ~70% sequence identity. Sequence alignment analysis was performed separately to evaluate their sequence conservation (Fig. 1j). The D loop of mascRNA is larger than that of menRNA, with the AC loop leaning more towards UUU. In contrast, the AC of menRNA is longer. Overall, they exhibit comparable conservation, suggesting a potential common evolutionary ancestor. Correspondingly, the tertiary structures of mascRNA and menRNA were well superimposed (Fig. 1h; Supplementary Fig. S3). In addition to the similarities in overall folding, the tertiary interactions that stabilize the L-shaped structure of menRNA resemble those of mascRNA. Apart from the elbow region (Fig. 1h, f), the base triplets G45-(*cis* Watson–Crick–Hoogsteen)-G8-(*cis* Watson–Crick)-C17 in menRNA are equivalent to the base triplets C16–G10–A44 in mascRNA (Fig. 1i), contributing to the resemblance in intramolecular interactions.

We further conducted a cross-species comparison of mascRNA between humans and medaka (Supplementary Fig. S5). Despite fewer GU base pairs in the AS and a more compact D loop due to the lack of a uracil nucleotide in medaka mascRNA, both structures superimpose well with each other with an RMSD of 2.2 Å (Supplementary Fig. S5a). In medaka mascRNA, the base triple is formed by a hydrogen bond donated from the N4 of C15 to the O4 of U43, coupled with the G10–C15 base pair of D stem, which is comparable to the C16–G10–A44 triple in human mascRNA (Supplementary Fig. S5b, c). Furthermore, the hydrogen bond donated from G11 N1 to U44 C6 is analogous to the interaction between U12 N3 and U45 O2 in human mascRNA. The high degree of structural similarity observed in mascRNAs between medaka and humans implies a strong selective pressure to maintain these elements.

Our research focused on unraveling the crystal structures of mascRNA and menRNA, both of which adopt a 3D “L”-shaped folding reminiscent of canonical tRNA. One key observation is the role of two evolutionarily conserved guanosines from the DSL in creating an “elbow” region along with the TSL, which is crucial for recognition by RNase P and RNase Z. Unlike tRNA, mascRNA has a reduced DSL and lacks certain critical tertiary interactions for stabilizing the canonical tRNA structure. Nevertheless, both mascRNA and menRNA exhibit notable structural homology and conserved elements, indicating a potential common evolutionary origin.

From a secondary structural perspective, the reduced size of mascRNA and menRNA has prompted some researchers to propose that these RNAs may superimpose more closely with the structure found in the tRNA-like domain of transfer-messenger RNA (tmRNA) (Supplementary Fig. S6). This resemblance suggests that the function of mascRNA and menRNA might necessitate assistance from proteins.

Moreover, the four-way junction, which is the second most common junction after the three-way junction, is known for its flexibility. It can adopt at least nine distinct tertiary structures, often compared to the letters H, L, K, W, and X, among others. Our crystallographic studies have revealed that mascRNA and menRNA have two coaxially stacked helical pairs that are oriented perpendicularly, forming an “L” shape. This characteristic places them in the same structural family as tRNA, as well as segments of the 16S and 23S rRNAs, as described elsewhere^6^.

Recent advancements in computational structural biology, notably the application of AlphaFold 3 have enabled the high-accuracy prediction of biological macromolecule structures^7^. Despite minor discrepancies in the predicted intramolecular interactions, AlphaFold 3 has provided reasonably accurate predictions of the structures of mascRNA and menRNA, serving as an auxiliary tool in structural determination (Supplementary Fig. S7).

During the maturation process of tRNA, both RNase P and RNase Z recognize the elbow region and measure the distance to identify the cleavage sites on pre-tRNA. The RNase P ribozyme uses a double-T-loop motif (IDTM) to identify the stacked bases at the pre-tRNA elbow (G19–C56), while the RNase Z protein recognizes the elbow through electrostatic interactions, hydrophobic effects, and shape complementarity (Supplementary Fig. S8)^8^. The high degree of structural similarity in the elbow region of these three RNAs explains the recognition of mascRNA and menRNA by RNase P and RNase Z. As a tRNA-like element, mascRNA is not aminoacylated. Our structure indicates that this is not solely due to the absence of a conserved anticodon loop in mascRNA. The deviation in the angle of the inner corner, along with the distance discrepancy between the AS and AC ends, also impedes aminoacylation^9^.

The evolutionarily conserved NEAT1 and MALAT1 genes have garnered significant interest in the fields of cardiovascular medicine and oncology. Recent years have witnessed an emerging body of research on the functions of mascRNA, which, upon binding to glutamyl-tRNA synthetase and heterogeneous nuclear ribonucleoprotein H/F (hnRNP H/F), can enhance protein translation, promote proliferation and metastasis, and modulate innate antiviral immunity^10^. However, the specific functions of menRNA are not fully understood, possibly due to its rapid degradation after the addition of a 3’ CCACCA sequence, making it challenging to detect using northern blot analysis. Furthermore, studying mascRNA modifications and its interactions with proteins could offer valuable insights into how it is recognized and how it carries out its functions.

In summary, our study offers a structural basis for understanding how mascRNA and menRNA mimic tRNA. This suggests a potential mechanism in which mimicry of the elbow region is crucial for recruiting RNase P and RNase Z, thereby facilitating their processing and maturation. These structural insights lay a foundation for further exploration of the biological roles of mascRNA and menRNA.

## Supplementary information

Supplementary Information

## Data Availability

The coordinates and structure factors of all the reported crystal structures have been deposited in the PDB under accession numbers 8K0Y (Medaka mascRNA U23G with iridium), 8K30 (Medaka mascRNA U23G), 8K1E (Human menRNA), and 8K2Z (Human mascRNA A2G).
