# Supplementary material for: Structural basis for tRNA mimicry by mascRNA and menRNA
Source: Cell Discov. 2025 Jan 2;10:128. doi: 10.1038/s41421-024-00761-1 (PMC11693755; doi:10.1038/s41421-024-00761-1)
Supplement: Supplementary file 1 — Supplementary Information [file 41421_2024_761_MOESM1_ESM.pdf]

Supplementary Information

Structural basis for tRNA mimicry by mascRNA and menRNA

Yuanlin He[1], Jie Deng[1], Xiaowei Lin[1,2,3], Zhizhong Lu[1,4], Liangliang Wang[5], Liang Xu[5], Yin Zhang[1,6], Jia Wang[7]* and Lin Huang[1]*

*Correspondence: Jia Wang (wangjia2@sztu.edu.cn) or
Lin Huang (huanglin36@mail.sysu.edu.cn)

These authors contributed equally: Yuanlin He, Jie Deng, Xiaowei Lin

[1]*Guangdong Provincial Key Laboratory of Malignant Tumor Epigenetics and Gene Regulation, Guangdong-Hong Kong Joint Laboratory for RNA Medicine, Sun Yat-Sen Memorial Hospital, Sun Yat-Sen University, Guangzhou, China*
[2]*Department of Urology, Sun Yat-Sen Memorial Hospital, Sun Yat-Sen University, Guangzhou, China*
[3]*Department of Urology, Dafeng Hospital, Chaoyang District, Shantou City, Guangdong Province, China*
[4]*School of Life Sciences and Biopharmaceutics, Guangdong Pharmaceutical University, Guangzhou, China*
[5]*MOE Key Laboratory of Bioinorganic and Synthetic Chemistry, School of Chemistry, Sun Yat-Sen University, Guangzhou, China*
[6]*Department of Cellular and Molecular Diagnostics Center, Sun Yat-Sen Memorial Hospital, Sun Yat-Sen University, Guangzhou, China*
[7]*College of Pharmacy, Shenzhen Technology University, Shenzhen 518118, China*

Materials and Methods

Supplementary Figures S1 – S8

Supplementary Table S1 – S2

Supplementary References

The input sequences for RNA secondary structure consensus analysis

The aligned sequences for RNA secondary structure consensus analysis

MATERIALS AND METHODS

Synthesis of RNA by transcription

DNA templates with a T7 RNA polymerase promoter were synthesized using the polymerase chain reaction (PCR) for in vitro transcription. The transcription process was carried out at 37°C for 5 to 6 hours. The resulting RNA was purified by 10% denaturing polyacrylamide gel electrophoresis in 7 M urea. After visualizing the gel under ultraviolet light, the specific RNA band was excised and subjected to electroelution (150V at 4°C for 5 hours) in a buffer solution containing 45 mM Tris-borate and 1 mM EDTA. The RNA was precipitated from the eluate using isopropanol, followed by a single wash with 75% ethanol. Finally, the purified RNA was redissolved in double-distilled water to a final concentration of 10 mg/ml, ready for crystallization studies.

Crystallization, structure determination and refinement

RNA samples (10 μg/μl) in 5 mM HEPES (pH 7.5), 100 mM KCl, and 5 mM $MgCl_2$ (referred to as HKM buffer) were heated to 95°C for 1 min, followed by gradual cooling at room temperature. Detailed RNA sequences and crystallization conditions are provided in **Supplementary Table S1**. Crystallization was achieved by combining 0.2 μl of the RNA solution with 0.2 μl of the reservoir solution using the sitting drop vapor diffusion method at 18°C. Well-diffracting crystals of mascRNA and menRNA (**Supplementary Table S1**) appeared and grew to full size throughout 2 to 5 days. To acquire derivatized crystals for phasing, Medaka mascRNA U23G was soaked in 12mM of iridium hexammine for 2 hours before harvesting. Subsequently, the crystals were transferred into a mother liquor supplemented with an additional 30% or 40% MPD, followed by rapid freezing through mounting onto nylon loops and immediate immersion in liquid nitrogen. X-ray diffraction data were collected on beamline BL10U2 or BL19U1 at Shanghai Synchrotron Radiation Facility (SSRF) and processed using XIA2 or DIALS[1]. The 2.28 Å structure deposited as 8K0Y was determined utilizing Ir-SAD via AutoSol in the PHENIX suite. The model was manually adjusted using Coot and subjected to several rounds of adjustment and optimization using Coot, phenix.refine, and PDB REDO[2]. All other structures were determined by molecular replacement using PHASER[3] with PDB 8K0Y. The translation function Z scores (TFZ) and log-likelihood gains (LLG) are reported in **Supplementary Table S1**. Model geometry and the fit to electron-density maps were monitored with MOLPROBITY and the validation tools in Coot. Simulated annealing omit maps were calculated based on the composite omit map in the PHENIX suite using the method anneal. Atomic coordinates and structure factor amplitudes have been submitted to the Protein

Data Bank (PDB) with the corresponding accession codes listed in **Supplementary Table S2**, together with the statistics of crystal diffraction data and structure refinement.

Gel electrophoretic analysis of D-T loop interaction

Polyacrylamide gel electrophoresis was used to assess the impact of disrupting the D-T loop interaction. The RNA hybridization involved denaturation at 95°C for 1 minute in a solution containing 25 mM Tris, 192 mM glycine (pH 8.3), and 1 mM $MgCl_2$, followed by gradual cooling. Electrophoresis was performed in 10% polyacrylamide gels at a constant power of 2W for 4 h at room temperature. Following electrophoresis, RNA was detected by UV shadowing. The sequences (all written 5' to 3') for native gel electrophoretic analysis were as follows:

Human mascRNA WT:
GAUGCUGGUGGUUGGCACUCCUGGUUUCCAGGACGGGGUUCAAAUCCCUGCGGCGUC

Human mascRNA DLM:
GAUGCUGGUGUUCGCACUCCUGGUUUCCAGGACGGGGUUCAAAUCCCUGCGGCGUC

Human mascRNA TLM:
GAUGCUGGUGGUUGGCACUCCUGGUUUCCAGGACGGGGUUCGCCCUGCGGCGUC

Human mascRNA G15C:
GAUGCUGGUGGUUGCCACUCCUGGUUUCCAGGACGGGGUUCAAAUCCCUGCGGCGUC

Human mascRNA G14C/G15C:
GAUGCUGGUGGUUCCCACUCCUGGUUUCCAGGACGGGGUUCAAAUCCCUGCGGCGUC

Human mascRNA C41U:
GAUGCUGGUGGUUGGCACUCCUGGUUUCCAGGACGGGGUUUAAAUCCCUGCGGCGUC

RNA consensus secondary structure analysis

The secondary structure of the conservative sequence analysis was performed by the R2R visualization program (version 1.0.6)[4]. R2R takes a multiple-sequence alignment in Stockholm format as input with additional annotation to direct the drawing. R2R software is

available at http://breaker.research.yale.edu/R2R. The input sequences and aligned sequences are provided in two separate files.

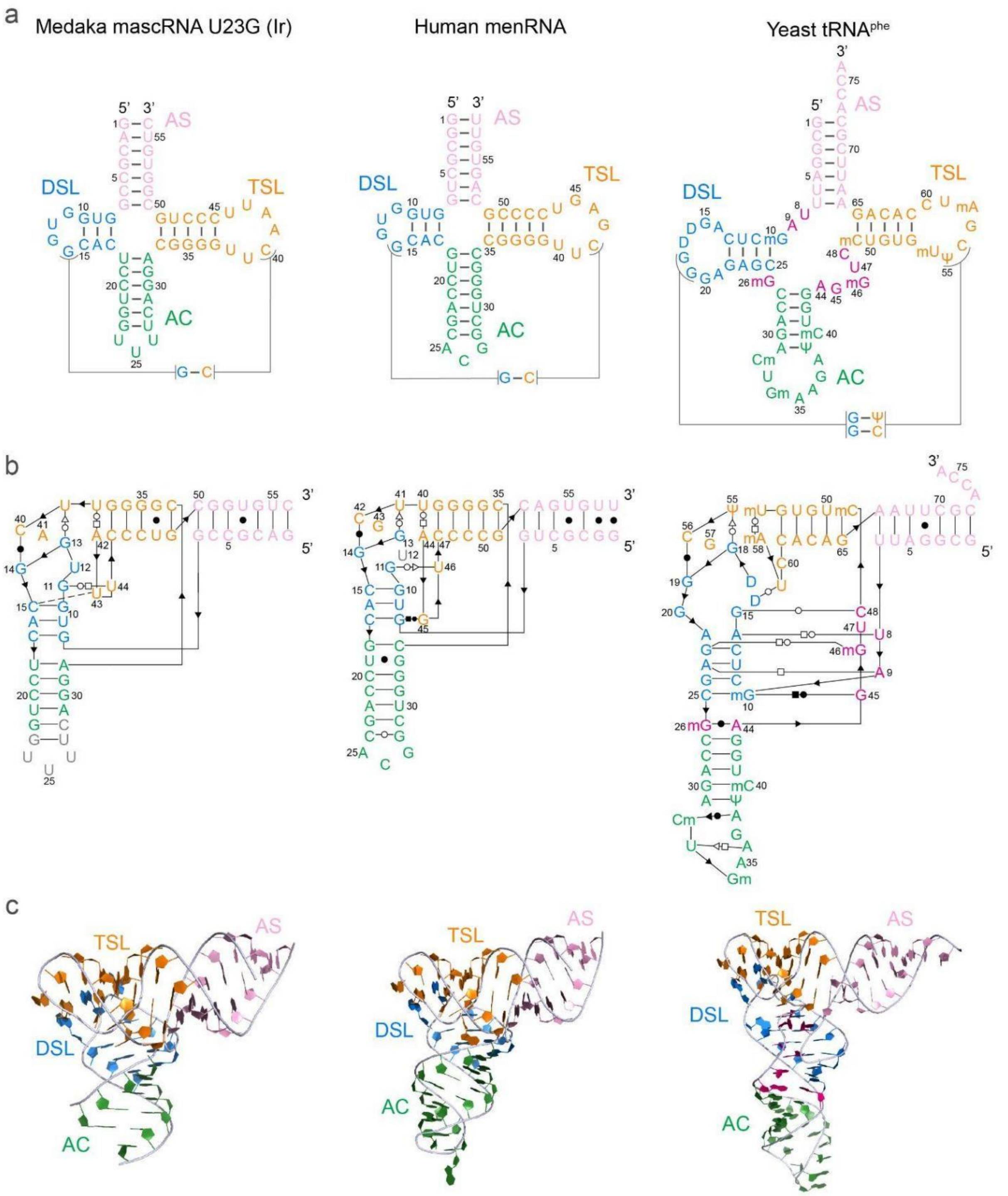
a
Medaka mascRNA U23G (lr)
Human menRNA
Yeast tRNAphe
AS
DSL
TSL
AC
b
c
TSL
AS
DSL
AC

Supplementary Fig. S1 Overall structure of medaka mascRNA and human menRNA. **a** Comparison between the secondary structures of medaka mascRNA, human menRNA and yeast $tRNA^{phe}$. Eight single-stranded linker residues absent from mascRNA are indicated in hotpink. (DSL: marine, AC: forest, TSL: orange, AS: pink). Long-range interactions between DSL and TSL are indicated. **b** Crystal structure–derived secondary structures of medaka mascRNA, human menRNA and yeast $tRNA^{phe}$. Base-pairing symbols follow Leontis–Westhof symbols. **c** Overall crystal structures of medaka mascRNA, human menRNA and yeast $tRNA^{phe}$ (PDB:1EHZ)[5], colored to match scheme used in **a**.

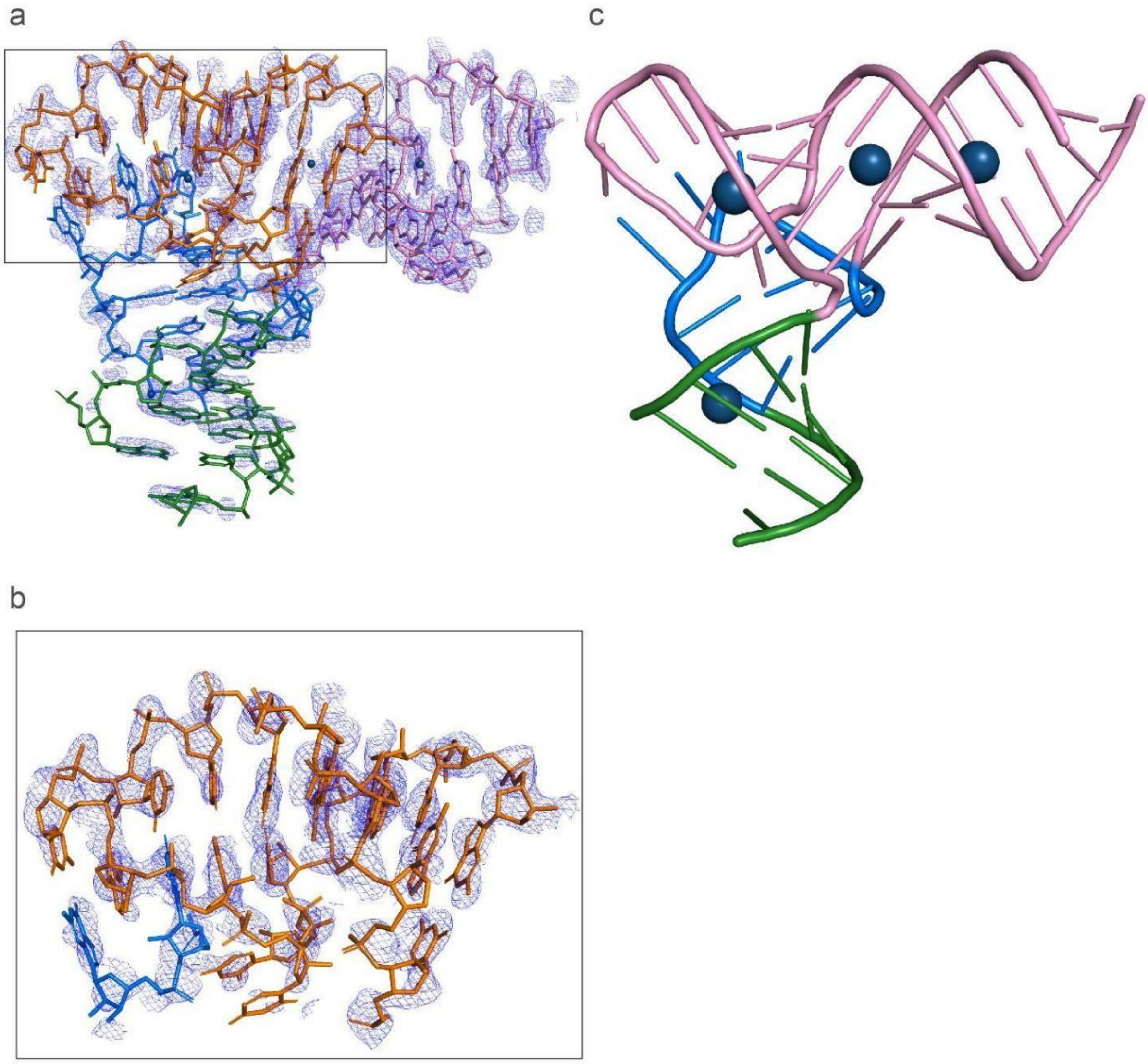

Supplementary Fig. S2 Representative electron density and bound ions of medaka mascRNA. **a** Unbiased, density-modified electron density from SAD phasing using data to 2.28 Å (light-blue mesh, 2σ), superimposed on the final model. The loop in AC is omitted. For simplicity, the density and structure of water is not shown. **b** The electron density of the TSL and G13, G14 from DSL. **c** Structure with the location of 4 iridium (III) hexammine ions, shown in blue spheres.

a

Overall superposition

RMSD	Human mascRNA 8K2Z	Medaka mascRNA lr 8K0Y	Medaka mascRNA 8K30	Human menRNA 8K1E	Yeast tRNA 1EHZ
Human mascRNA 8K2Z (1-57)	0				
Medaka mascRNA lr 8K0Y (1-56)	2.231	0			
Medaka mascRNA 8K30 (1-56)	1.832	1.224	0		
Human menRNA 8K1E (1-58)	4.431	3.682	3.805	0	
Yeast tRNA 1EHZ (1-72)	13.254	15.516	15.087	10.560	0

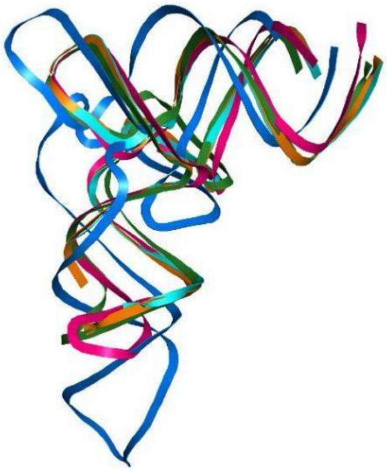

b

AS+TSL+GG

RMSD	Human mascRNA 8K2Z	Medaka mascRNA lr 8K0Y	Medaka mascRNA 8K30	Human menRNA 8K1E	Yeast tRNA 1EHZ
Human mascRNA 8K2Z (14-15, 1-7, 34-57)	0				
Medaka mascRNA lr 8K0Y (13-14, 1-7, 33-56)	1.875	0			
Medaka mascRNA 8K30 (13-14, 1-7, 33-56)	1.650	0.677	0		
Human menRNA 8K1E (13-14, 1-7, 35-58)	2.430	1.478	1.364	0	
Yeast tRNA 1EHZ (18-19, 1-7, 49-72)	15.519	4.703	4.805	6.539	0

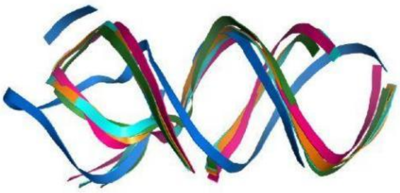

c

AS+TSL

RMSD	Human mascRNA 8K2Z	Medaka mascRNA lr 8K0Y	Medaka mascRNA 8K30	Human menRNA 8K1E	Yeast tRNA 1EHZ
Human mascRNA 8K2Z (1-7, 34-57)	0				
Medaka mascRNA lr 8K0Y (1-7, 33-56)	1.739	0			
Medaka mascRNA 8K30 (1-7, 33-56)	1.496	0.689	0		
Human menRNA 8K1E (1-7, 35-58)	2.287	1.448	1.346	0	
Yeast tRNA 1EHZ (1-7, 49-72)	13.033	10.545	10.490	13.550	0

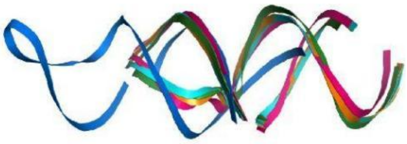

d

TSL+GG

RMSD	Human mascRNA 8K2Z	Medaka mascRNA lr 8K0Y	Medaka mascRNA 8K30	Human menRNA 8K1E	Yeast tRNA 1EHZ
Human mascRNA 8K2Z (14-15, 34-50)	0				
Medaka mascRNA lr 8K0Y (13-14, 33-49)	0.981	0			
Medaka mascRNA 8K30 (13-14, 33-49)	0.776	0.698	0		
Human menRNA 8K1E (13-14, 35-51)	1.138	1.176	0.973	0	
Yeast tRNA 1EHZ (18-19, 49-65)	2.632	1.349	1.247	2.693	0

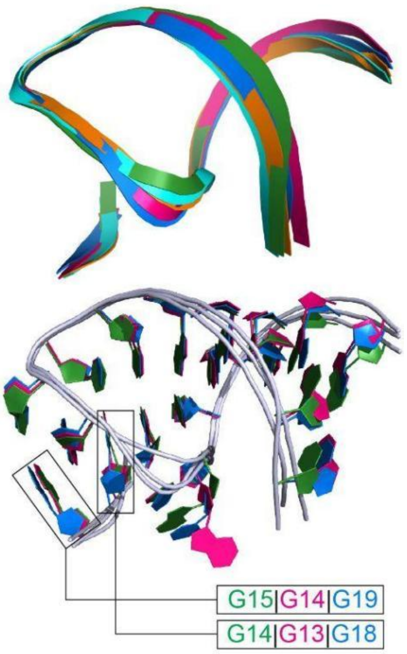

e

TSL

RMSD	Human mascRNA 8K2Z	Medaka mascRNA lr 8K0Y	Medaka mascRNA 8K30	Human menRNA 8K1E	Yeast tRNA 1EHZ
Human mascRNA 8K2Z (34-50)	0				
Medaka mascRNA lr 8K0Y (33-49)	0.956	0			
Medaka mascRNA 8K30 (33-49)	0.674	0.716	0		
Human menRNA 8K1E (35-51)	1.121	1.174	0.960	0	
Yeast tRNA 1EHZ (49-65)	2.698	1.265	1.201	2.877	0

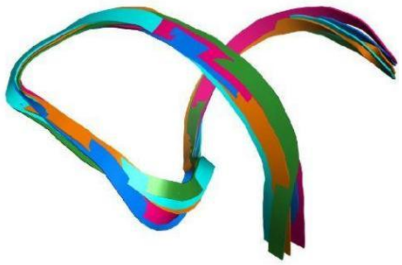

f

Superposition based on TSL GG

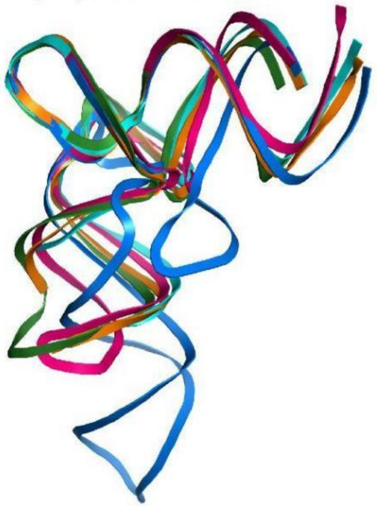

Supplementary Fig. S3 All-atom RMSD analysis of different regions of mascRNA, menRNA and tRNA. **a, b, c, d, e** RMSD (left) and structural overlay (right) of overall superposition (**a**), AS + TSL + GG superposition (**b**), AS + TSL superposition (**c**), TSL + GG superposition (**d**) and TSL superposition (**e**) along human mascRNA (forest), medaka mascRNA lr (cyan), medaka mascRNA (orange), human menRNA (hotpink) and yeast $tRNA^{phe}$ (marine). The PDB of the structures are shown in dark red and the numbers in parentheses refer to the base for superposition. All-atom RMSD was calculated by employing the "align" function in PyMOL, with the cycles parameter set to zero. The RMSD values over 5 are marked in bright red. **f** Structural superpositions of human mascRNA, medaka mascRNA, human menRNA and yeast $tRNA^{phe}$, superimposed on the TSL and GG.

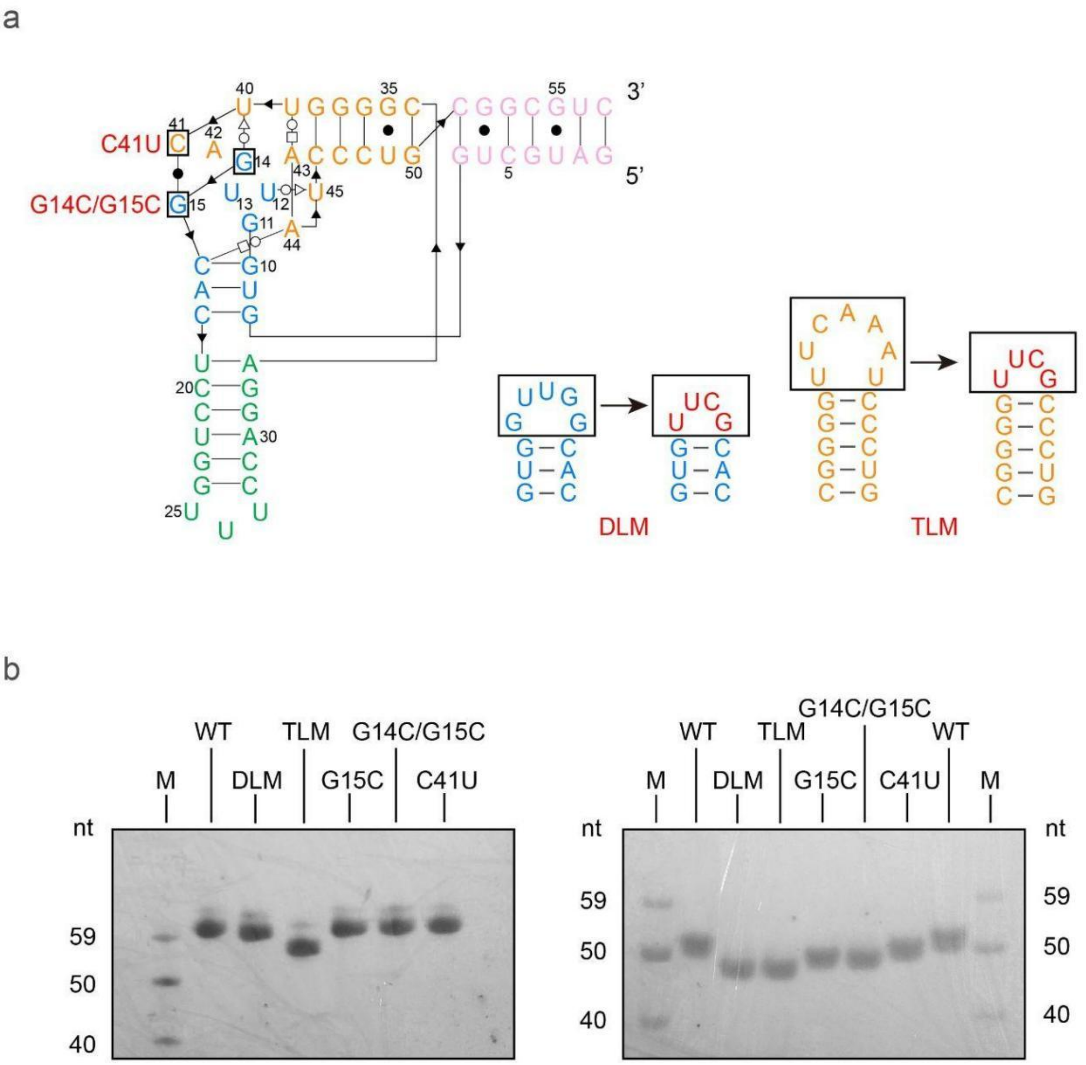

Supplementary Fig. S4 Effect of breaking the D-T loop interaction. **a** Diagram of mutations in Human mascRNA. The black boxes indicate mutated bases. **b** The urea polyacrylamide gel electrophoresis (uPAGE) (left) and native polyacrylamide gel electrophoresis (nPAGE) (right) analysis of WT (57nt), D loop mutant (DLM, 56nt), T loop mutant (TLM, 54nt), G15C (57nt), G14C/G15C (57nt), and C41U (57nt). “M” represents the marker.

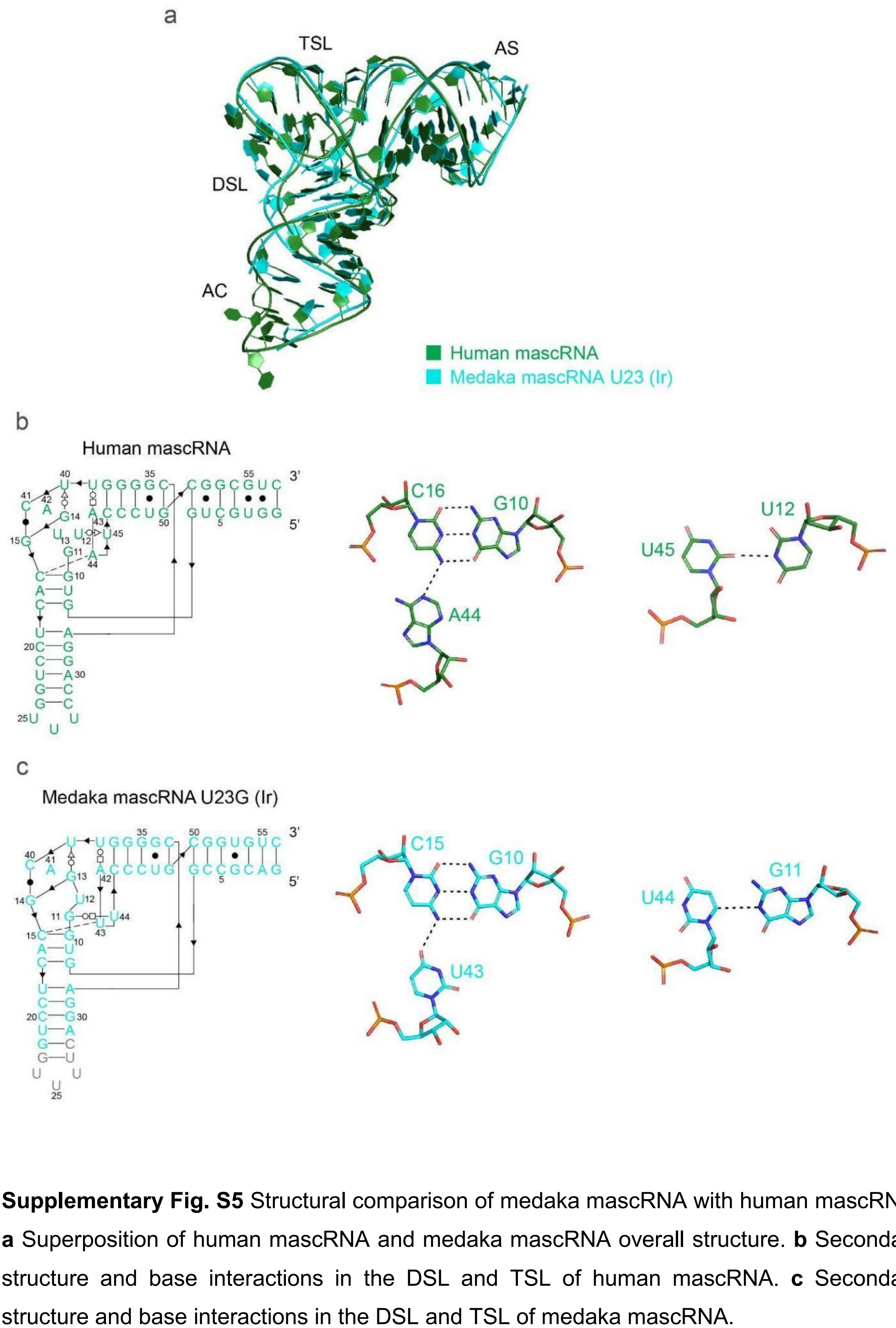

Supplementary Fig. S5 Structural comparison of medaka mascRNA with human mascRNA. **a** Superposition of human mascRNA and medaka mascRNA overall structure. **b** Secondary structure and base interactions in the DSL and TSL of human mascRNA. **c** Secondary structure and base interactions in the DSL and TSL of medaka mascRNA.

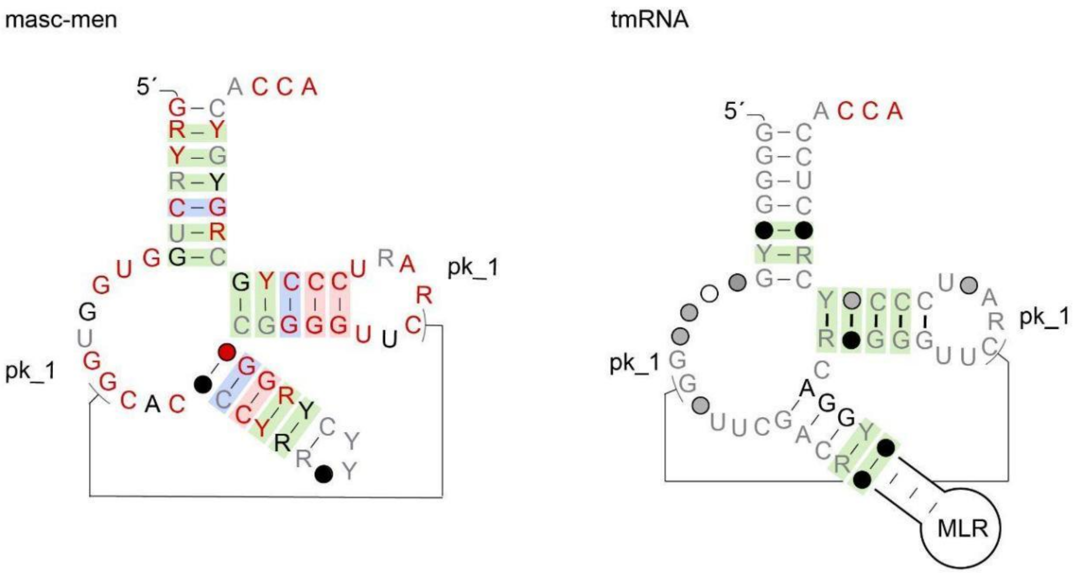

Supplementary Fig. S6 The secondary structure of mascRNA and menRNA, the second possibility—"tmRNA-like", visualized using the R2R program[6]**.** The figure highlights their conserved areas. Helix regions are indicated by continuous stretches of base pairs. Pseudoknots (pk), which are represented by the interlocking loops (such as pk_1), show regions where the RNA folds back on itself, forming additional base pairs that are not part of the helical regions.

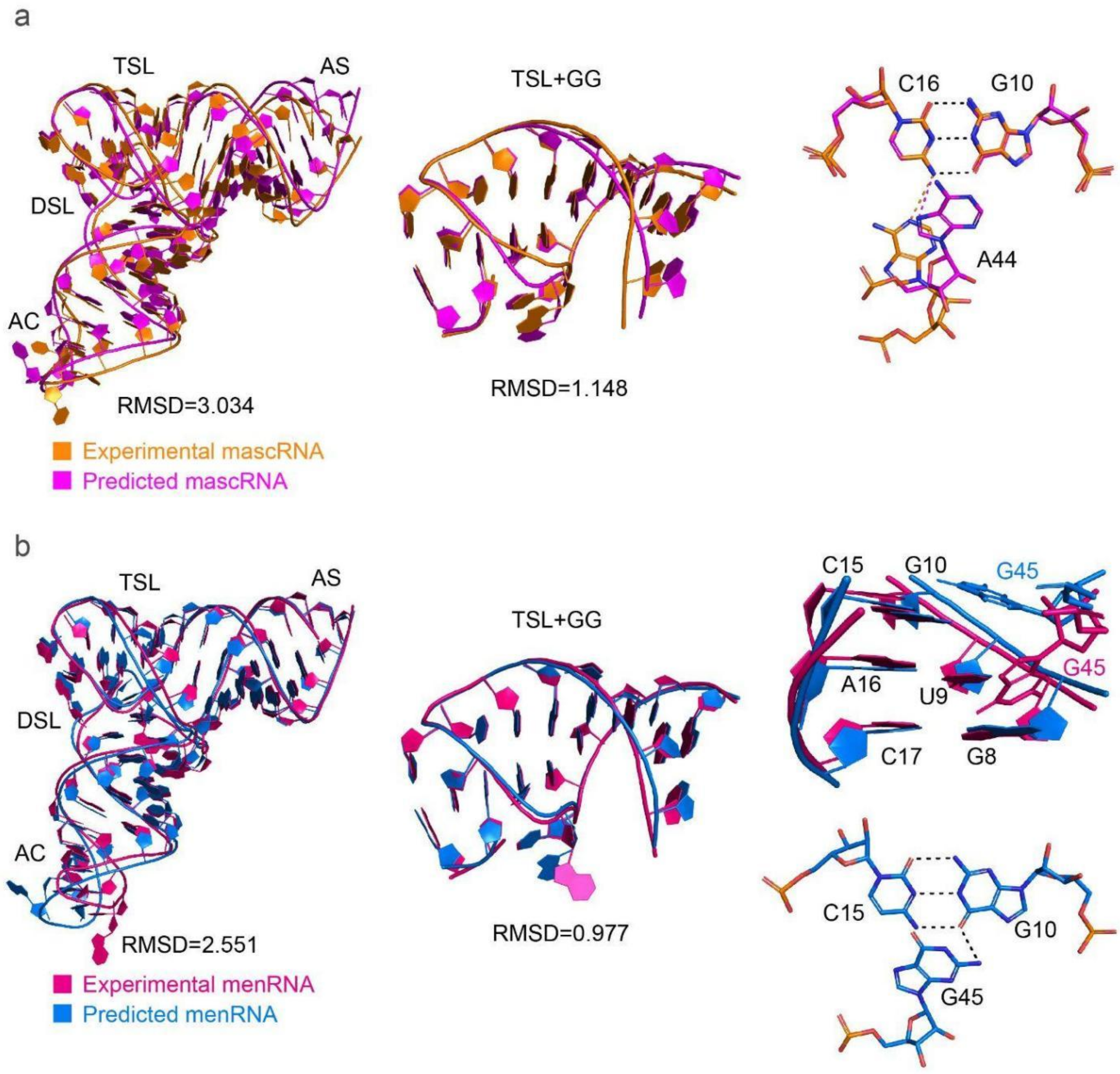

Supplementary Fig. S7 Comparison of experimental structure and predicted structure by AlphaFold 3. **a** Superposition of experimental and predicted human mascRNA structures, focusing on the overall, TSL+GG and base triple regions. The predictive model with the highest ranking score is selected for comparative analysis and RMSD analysis is based on all atoms. **b** Superposition of experimental and predicted human menRNA structures, focusing on the overall, TSL+GG and base triple regions.

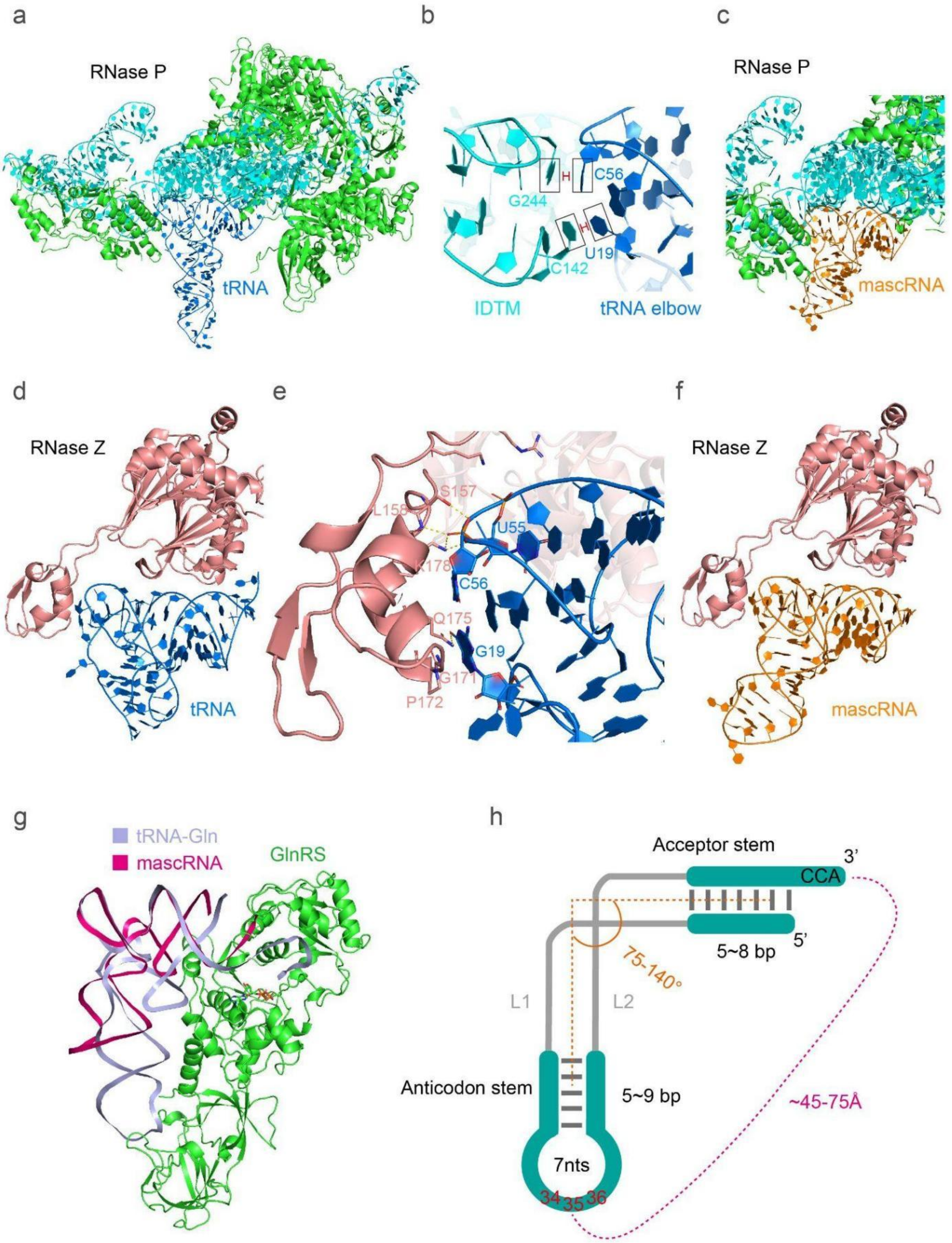
a
RNase P
tRNA
b
C56
G244
U19
C142
IDTM
tRNA elbow
c
RNase P
mascRNA
d
RNase Z
tRNA
e
S157
L158
U55
K178
C56
Q175
G19
G171
P172
f
RNase Z
mascRNA
g
tRNA-Gln
mascRNA
GlnRS
h
Acceptor stem
3'
CCA
5'
5~8 bp
75-140°
L1
L2
Anticodon stem
5~9 bp
~45-75Å
7nts
34
35
36

Supplementary Fig. S8 Enzymes recognizing tRNA interact with its interface, indicating mascRNA fate. **a** Overall structure of the human RNase P in complex with $tRNA^{Val}$ (PDB:6AHU)[7]. **b** Detailed stacking interfaces at the tRNA elbow that are recognized by the IDTM of RNase P. **c** Simulated interaction between RNase P and mascRNA elbow based on tRNA-mascRNA structural superposition. **d** Overall structure of the Bacillus subtilis RNase Z in complex with $tRNA^{Thr}$ (PDB:2FK6)[8]. **e** Detailed interfaces at the tRNA elbow that are recognized by RNase Z. **f** Simulated interaction between RNase Z and mascRNA elbow. **g** Superposition of human mascRNA to the glutamyl-tRNA (tRNA-Gln) bound to glutamyl-tRNA synthetase (GlnRS)[9]. **h** Structural prerequisites essential for the functionality of tRNAs recognized by aminoacyl-tRNA synthetases (AARS)[10]. Diverse orientations of the two stems are observed, with the interval between the CCA_{OH} end and the anticodon triplet extending from 45 to 75 Å.

Supplementary Table S1 Sequences and conditions employed in crystallization trials. SAD-Ir: single-wavelength anomalous dispersion method with iridium hexamine soaking. MR: molecular replacement.

PDB	Crystallized with	Mother liquor	Cryoprotectant	Res	SG	Phasing
8K0Y	Single stranded RNA	0.08 M Strontium chloride hexahydrate<br>0.02 M Magnesium chloride hexahydrate<br>0.04 M Sodium cacodylate trihydrate pH 7.8<br>18% v/v (+/-)-2-Methyl-2,4-pentanediol<br>0.012 M Spermine tetrahydrochloride<br>Soak in 12mM iridium for 2 hours	40% MPD	2.28	C 1 2 1	SAD-Ir
Medaka mascRNA U23G (soaking in iridium)	GACGCCGGUGGUGGCACUCCUGGUUUUCAGGACGGGGUUCAAUUCCCUGCGGUGUC					56nt
WT	GACUCCGGUGGUGGCACUCCUGUUUUACUCAGGACGGGGUUCAAUUCCCUGCGGUGUCUUGC					62nt
8K30	Single stranded RNA	0.08 M Strontium chloride hexahydrate<br>0.02 M Magnesium chloride hexahydrate<br>0.04 M Sodium cacodylate trihydrate pH 8.6<br>18% v/v (+/-)-2-Methyl-2,4-pentanediol<br>0.012 M Spermine tetrahydrochloride	30% MPD	2.43	C 1 2 1	MR<br>Model:8K0Y<br>TFZ=29.8<br>LLG=2664.2
Medaka mascRNA U23G	GACGCCGGUGGUGGCACUCCUGGUUUUCAGGACGGGGUUCAAUUCCCUGCGGUGUC					56nt
WT	GACUCCGGUGGUGGCACUCCUGUUUUACUCAGGACGGGGUUCAAUUCCCUGCGGUGUCUUGC					62nt
8K1E	Single stranded RNA	0.04 M Lithium chloride<br>0.08 M Strontium chloride hexahydrate<br>0.02 M Magnesium chloride hexahydrate<br>0.04 M Sodium cacodylate trihydrate pH 7.0<br>28% v/v (+/-)-2-Methyl-2,4-pentanediol<br>0.012 M Spermine tetrahydrochloride	30% MPD	2.23	C 1 2 1	MR<br>Model:8K0Y<br>TFZ=53<br>LLG=5584
Human menRNA	GGCGCUGGUGGUGGCACGUCCAGCACGGCUGGGCCGGGGUUCGAGUCCCCGCAGUGUU					58nt
WT	GGCGCUGGUGGUGGCACGUCCAGCACGGCUGGGCCGGGGUUCGAGUCCCCGCAGUGUUGCUGC					63nt
8K2Z	Single stranded RNA	0.08 M Strontium chloride hexahydrate<br>0.02 M Magnesium chloride hexahydrate<br>0.04 M Sodium cacodylate trihydrate pH 8.0<br>18% v/v (+/-)-2-Methyl-2,4-pentanediol<br>0.012 M Spermine tetrahydrochloride	Direct	2.40	P 1 2_1 1	MR<br>Model:8K0Y<br>TFZ=9.9<br>LLG=397
Human mascRNA A2G	GGUGCUGGUGGUUGGCACUCCUGGUUUCCAGGACGGGGUUCAAAUCCCUGCGGCGUC					57nt
WT	GAUGCUGGUGGUUGGCACUCCUGGUUUCCAGGACGGGGUUCAAAUCCCUGCGGCGUCUCCA					61nt

Supplementary Table S2 Details of data collection and refinement statistics for the crystallographic data as deposited with the PDB.

Name	Medaka mascRNA U23G （soaking in iridium）	Medaka mascRNA U23G	Human menRNA	Human mascRNA A2G
PDB	8K0Y	8K30	8K1E	8K2Z
**Data collection**				
Space group	C 1 2 1	C 1 2 1	C 1 2 1	P 1 2_1 1
Cell dimensions				
a, *b*, *c* (Å)	159.4, 29.3, 98.1	157.9, 29.2, 99.1	137.6, 29.2, 99.1	79.3, 30.1, 89.4
α, β, γ (°)	90 119 90	90 118 90	90 127 90	90 106 90
Phasing	SAD-Ir	MR	MR	MR
Wavelength	0.9792	0.9792	0.9792	0.9785
Resolution (Å)	28.72 – 2.28 (2.34 – 2.28)	37.02 – 2.43 (2.49 – 2.43)	79.44 – 2.23 (2.29 – 2.23)	66.76 – 2.40 (2.46 – 2.40)
R_{merge} (within I+/I-)	0.079 (1.174)	0.054 (0.813)	0.086 (0.921)	0.095 (0.783)
Rpim (within I+/I-)	0.052 (0.802)	0.036 (0.641)	0.057 (0.751)	0.061 (0.520)
$I / \sigma I$	12.4 (1.6)	11.5 (1.5)	9.7 (1.8)	7.8 (1.7)
CC (1/2)	1.00 (0.62)	1.00 (0.82)	0.90 (0.37)	1.00 (0.50)
Completeness (%)	99.6 (99.8)	99.9 (100)	99.2 (90.3)	99.9 (100)
Redundancy	6.1 (5.5)	5.9 (4.7)	5.5 (3.4)	6.2 (6.2)
**Refinement**				
Resolution (Å)	28.08 – 2.28 (2.36 – 2.28)	31.08 – 2.43 (2.52 – 2.43)	34.15 – 2.23 (2.31 – 2.23)	28.67 – 2.40 (2.49 – 2.40)
No. reflections	18653 (1810)	15594 (1499)	15785 (1413)	16464 (1642)
R_{work} / R_{free}	0.249 / 0.285	0.264 / 0.284	0.236 / 0.266	0.280 / 0.295
No. atoms				
macromolecules	2141	2204	2385	2320
ligands	20	13	16	12
solvent	14	11	82	24
B-factors				
macromolecules	87.18	88.98	54.49	92.72
ligands	87.78	68.97	43.69	57.30
solvent	56.86	61.65	40.68	54.12
R.m.s. deviations				
Bond lengths (Å)	0.005	0.005	0.002	0.005
Bond angles (°)	0.96	1.07	0.59	1.16

*Values in parentheses are for the highest-resolution shell.

SUPPLEMENTARY REFERENCES

1. Winter, G. *et al.* DIALS: implementation and evaluation of a new integration package. *Acta Crystallogr D Struct Biol* **74**, 85–97 (2018).
2. Joosten, R. P., Long, F., Murshudov, G. N. & Perrakis, A. The PDB_REDO server for macromolecular structure model optimization. *IUCrJ* **1**, 213–220 (2014).
3. McCoy, A. J. *et al.* Phaser crystallographic software. *J. Appl. Crystallogr.* **40**, 658–674 (2007).
4. Weinberg, Z. & Breaker, R. R. R2R--software to speed the depiction of aesthetic consensus RNA secondary structures. *BMC Bioinformatics* **12**, 3 (2011).
5. Shi, H. & Moore, P. B. The crystal structure of yeast phenylalanine tRNA at 1.93 A resolution: a classic structure revisited. *RNA* **6**, 1091–1105 (2000).
6. Weis, F. *et al.* tmRNA-SmpB: a journey to the centre of the bacterial ribosome. *EMBO J.* **29**, 3810–3818 (2010).
7. Wu, J. *et al.* Cryo-EM Structure of the Human Ribonuclease P Holoenzyme. *Cell* **175**, 1393–1404.e11 (2018).
8. Li de la Sierra-Gallay, I., Mathy, N., Pellegrini, O. & Condon, C. Structure of the ubiquitous 3' processing enzyme RNase Z bound to transfer RNA. *Nat. Struct. Mol. Biol.* **13**, 376–377 (2006).
9. Rould, M. A., Perona, J. J. & Steitz, T. A. Structural basis of anticodon loop recognition by glutaminyl-tRNA synthetase. *Nature* **352**, 213–218 (1991).
10. Giegé, R. & Eriani, G. The tRNA identity landscape for aminoacylation and beyond. *Nucleic Acids Res.* **51**, 1528–1570 (2023).

The input sequences for RNA secondary structure consensus analysis

masc_Human
GAUGCUGGUGGUGGCACUCCUGGU.UU..CCAGGACGGGGUUCAAAUCCCUGC
GGCGUCACCA

masc_Chim
GAUGCUGGUGGUGGCACUCCUGGU.UU..CCAGGACAGGGUUCAAAUCCCUGC
GGCGUCACCA

masc_Goril
GAUGCUGGUGGUGGCACUCCUGGU.UU..CCAGGACGGGGUUCAAAUCCCUGC
GGCGUCACCA

masc_Orang
GAUGCUGGUGGUGGCACUCCUGGU.UU..CCAGGACGGGGUUCAAAUCCCUGC
GGCGUCACCA

masc_Gibb
GAUGCUGGUGGUGGCACUCCUGGU.UU..CCAGGACGGGGUUCAAAUCCCUGC
GGCGUCACCA

masc_Rhe
GAUGCUGGUGGUGGCACUCCUGGU.UU..CCAGGACGGGGUUCAAAUCCCUGC
GGCAUCACCA

masc_Crabe
GAUGCUGGUGGUGGCACUCCUGGU.UU..CCAGGACGGGGUUCAAAUCCCUGC
GGCAUCACCA

masc_Babo
GAUGCUGGUGGUGGCACUCCUGGU.UU..CCAGGACGGGGUUCAAAUCCCUGC
GGCAUCACCA

masc_Gree
GAUGCUGGUGGUGGCACUCCUGGU.UU..CCAGGACGGGGUUCAAAUCCCUGC
GGCAUCACCA

masc__Mar
GAUGCUGGUAGUGGUACUCCUGGU.UU..CCAGGACGGGGUUCAAAUCCCUGC
GGCAUCACCA

masc_Squirr
GAUGCUGGUGGUGGCACUCCUGGU.UU..CCAGGACGGGGUUCAAAUCCCUGC
GGCAUCACCA

masc__Bus
GAUGCUGGUGGUGGCACUCCUGGU.UU..CCAGGACGGGGUUCAAAUCCCUGU
GGCGUCACCA

masc__Chine
GAUGCUGGUGGUGGCACUCCUGGU.UC..ACAGGAUGGGGUUCAAAUCCCUAC
GGCAUCACCA

masc__Squir
GAUGCUGGUGGUGGCACUCCUGGC.UU..CCAGGACGGGGUUCAAAUCCCUGC
AGCGUCACCA

masc_Les
GAUGCUGGUGGUGGCACUCCUGGU.UU..CCAGGAUGGGGUUCAAAUCCCUGC
GGCAUCACCA

masc__Prair
GAUGCCGGUGGUGGCACUCCUGGU.UU..CCAGGACGGGGUUCAAAUCCCUGC
GGUGUCACCA

masc_Chines
GACACUGGUGGCGGCACUCCUGGU.CU..CCAGGACGGGGUUCAAAUCCCUGC
GGUGUCACCA

masc_Golden
GAUGCUGGUGGUGGCACUCCUGGU.UU..CCAGGACGGGGUUCAAAUCCCUGC
GGUGUCACCA

masc_Mouse
GACGCUGGUGGUGGCACUCCUGGU.UU..CCAGGACGGGGUUCAAGUCCCUGC
GGUGUCACCA

masc_Rau
GAUGCUGGUGGUGGCACUCCUGGU.UU..CCAGGACGGGGUUCAAGUCCCUGC
GGCGUCACCA

masc_Naked
GAUGCUGGUGGUGGCACUCCUGGU.UU..CCAGGACGGGGUUCAAAUCCCUGC
GGCGUCACCA

masc_Guine
GAUGCUGGUGGUGGCACUCCUGGU.UU..CCAGGACGGGGUUCAAAUCCCUGC
AGUGUCACCA

masc_Chinch
GAUGCUGGUGGUGGCACUCCUGGU.UU..CCAGGACGGGGUUCAAAUCCCUGC
GGCGUCACCA

masc_Brush
GAUGCUGGUGGUGGCACUCCUGGU.UU..CCAGGACGGGGUUCAAAUCCCUGC
AGCGUCACCA

masc_Rabbiu
GAUGCUGGUGGUGGCACUCCUGGA.UU..CCAGGACGGGGUUCAAAUCCCUGC
GGCGUCACCA

masc_Pika
GAUGCUGGUGGUGGCACUCCUGGU.CU..CCAGGACAGGGUUCAAAUCCCUGC
GGCGUCACCA

masc_Pig
GAUGCUGGUGGUGGCACUCCUGGC.UU..CCAGGACAGGGUUCAAAUCCCUGC
GGCGUCACCA

masc_Alpac
GAUGCUGGUGGUGGCACUCCUGGU.UU..CCAGGACAGGGUUCAAAUCCCUGU
GGCAUCACCA

masc_Bacuri
GAUGCUGGUGGUGGCACUCCUGGU.UU..CCAGGACAGGGUUCAAAUCCCUGU
GGCAUCACCA

masc_Dolphi
GAUGCUGGUGGUGGCACUCCUGGU.UU..CCAGGACGGGGUUCAAAUCCCUGU
GGCAUCACCA

masc_Killer
GAUGCUGGUGGUGGCACUCCUGGU.UU..CCAGGACGGGGUUCAAAUCCCUGU
GGCAUCACCA

masc_Uibeu
GAUGCUGGUGGUGGCACUCCUGGU.UU..CCAGGACGGGGUUCGAAUCCCUGU
GGCGUCACCA

masc_Cow
GAUGCUGGUGGUGGCACUCCUGGU.UU..CCAGGACGGGGUUCAAAUCCCUGU
GGCGUCACCA

masc_Shee
GAUGCUGGUGGUGGCACUCCUGGU.UU..CCAGGACGGGGUUCGAAUCCCUGU
GGCGUCACCA

masc_Dome
GAUGCUGGUGGUGGCACUCCUGGU.UU..CCAGGACGGGGUUCGAAUCCCUGU
GGCGUCACCA

masc_Hors
GAUGCUGGUGGUGGCACUCCUGGU.UU..CCAGGACGGGGUUCGAAUCCCUGC
GGCGUCACCA

masc_Whiue
GAUGCUAGUGAUGGCACUCCUGGU.UU..CCAGGACAGGGUUCAAAUCCCUGU
GGCGUCACCA

masc_Cau
GAUGCUGGUGGUGGCACUCCUGGU.UU..CCAGGACAGGGUUCAAAUCCCUGC
GGCGUCACCA

masc_Dog
GAUGCUGGUGGUGGCACUCCUGGAUUU..CCAGGACAGGGUUCAAAUCCUUG
CAGUGUCACCA

masc_Ferre
GAUGCUGGUGGUGGCACUCCUGAU.UC..CCAGGACGGGGUUCAAAUCCCUGC
GGUGUCACCA

masc_Pand
GAUGCUGGUGGUGGCACUCCUGA..UU..CCAGGACAGGGUUCAAAUCCCUGCG
GCGUCACCA

masc_Pacifi
GAUGCUGGUGGUGGCACUCCUGGU.UU..CCGGGACGGGGUUCAAAUCCCUGC
GGCGUCACCA

masc_Wedde
GAUGCUGGUGGUGGCACUCCUGGU.UU..CCAGGACAGGGUUCAAAUCCCUGC
GGCGUCACCA

masc_Black
GAUGCUGGUGGUGGCACUCCUGGU.UU..CCAGGACGGGGUUCAAAUCCCUGU
GGCGUCACCA

masc_Megaba
GAUGCUGGUGGUGGCACUCCUGGU.UU...CAGGACGGGGUUCAAAUCCGUGU
GGCGUCACCA

masc_Big_br
GAUGCUGGUGGUGGCACUCCUGGU.UU..CCAGGACAGGGUUCGAGUCCCUGU
GGCGUCACCA

masc_David'
GACGCUGGUGGUGGCACUCCUGGU.UU..CCAGGACAGGGUUCGAGUCCCUGU
GGCGUCACCA

masc_Liuule
GAUGCUGGUGGUGGCACUCCUGGU.UU..CCAGGACGGGGUUCGAGUCCCUGU
GGCGUCACCA

masc_Hedge
GAUGCUGGUGGUGGCACUCCUGAC.UU..UCAGGACAGGGUUCGAGUCCCUGC
GGUGUCACCA

masc_Shr
GAUGCUGGUGGUGGCACUCCUGGU.UU..CCAGGACAGGGUUCAAGUCCCUGC
GGUGUCACCA

masc_Suar
GAUGCUGGUGGUGGCACUCCUGGU.UG..CCAGGACGGGGUUCGAAUCCCUGC
GGCGUCACCA

masc_Elep
GAUGCUGGUGGUGGCACUCCUGUU.UU..CCAGGACGGGGUUCAAAUCCCUGC
AGCGUCACCA

masc_Cape
GAUGCUGGUGGUGGCACUCCUGGU.UU..CCAGGACGGGGUUCAAAUCCCUGC
GGCGUCACCA

masc_Manau
GAUGCUGGUGGUGGCACUCCUGUU.UU..CCAGGAUGGGGUUCAAAUCCCUGC
AGCGUCACCA

masc_Cap
GAUGCUGGUGGUGGCACUCCUGGG.UU..CCAGGACGGGGUUCAAAUCCCUGC
GGCGUCACCA

masc_Uenr
GAUGCUGGUGGUGGCACUCCUGGU.UC..CCAGGACGGGGUUCAAAUCCCUGC
AGUGUCACCA

masc_Aardva
GACGCUGGUGGUGGCACUCCUGGU.UU..CCAGGACGGGGUUCAAAUCCCUGC
GGCGUCACCA

masc_Arma
GAUGCUGGUGGUGGCACUCCUGGU.UU..CCAGGACGGGGUUCAAAUCCCUGU
GGCGUCACCA

masc_Opos
GAUACUGGUGGUGGCACUCCUGGC..U..CCAGGACAGGGUUCGAGUCCCUGCA
GUGUCACCA

masc_Uasma
GAUACUGGUGGUGGCACUCCUGGU.CU..CCGGGAUGGGGUUCGAGUCCCUAC
GGUGUCACCA

masc_Wall
GAUACUGGUGGUGGCACUCCUGGG.CU..CCAGGACGGGGUUCAAAUCCCUGC
GGUAUCACCA

masc_Plauyp
GAUGCUGGUGGUGGCACUCCUGGG.UU..CCAGGACAGGGUUCAAAUCCCUGC
AGUGUCACCA

masc_Chicke
GGCGCCGGUGGUGGCACUCCCUGU.......GGGACGGGGUUCGAAUCCCCGCGG
CGCCACCA

masc__Ameri
GAUGCUGGUGGUGGCACUCCUGGC.CU..CCAGGAUGGGGUUCAAGUCCCUAC
AGUGUCACCA

masc_Green
GAUGCUGGUGGUGGCACUCCUGGU.UU..CCAGGAUGGGGUUCAAAUCCCUAC
GGUGUCACCA

masc_Painue
GAUGCUGGUGGUGGCACUCCUGGU.UU..CCAGGAUGGGGUUCAAAUCCCUAC
AGUGUCACCA

masc_Chine
GAUGCUGGUGGUGGCACUCCUGGU.UU..CCAGGAUGGGGUUCAAAUCCCUGC
AGUGUCACCA

masc_Spi
GAUGCUGGUGGUGGCACUCCUGGU.UU..CCAGGAUGGGGUUCAAAUCCUUGC
AGUGUCACCA

masc__Liz
GACGCUGGUGGUGGCACUCCUGGC.UU..CCGGGACGGGGUUCAAGUCCCUGC
GGUGUCACCA

masc_X._ur
GGCACCGGUGGUGGCACACCUGAU.AU..UCAGGACGGGGUUCAAAUCCCUGC
GGUGUCACCA

masc_Coela
GAUACUGGUGGUGGCACUCCUGAU.UU..CCAGGACAGGGUUCGAUUCCCUGC
GGUGUCACCA

masc_Nile_
GACUCCGGUGGUGGCACUCCUGAC.UCUGUCGGGACGGGGUUCAAUUCCCUG
CGGUGUCACCA

masc_Buruon
GACUCCGGUGAUGGCACUCCUGAC.UCUGUCGGGACGGGGUUCAAUUCCCUG CGGUGUCACCA

masc_Pundam
GACUCCGGUGAUGGCACUCCUGAC.UCUGUCGGGACGGGGUUCAAUUCCCUG CGGUGUCACCA

masc_Medak
GACUCCGGUGGUGGCACUCCUGUU.UUACUCAGGACGGGGUUCAAUUCCCUG CGGUGUCACCA

masc_Souuhe
GACUCCGGUGGUGGCACUCCUGAC.CUGGUCGGGACGGGGUUCAAAUCCCUG CGGUGUCACCA

masc_Suickl
GACUCCGGUGGUGGCACUCCUGAC.UCUGUCAGGACGGGGUUCAAUCCCCUG CGGUGUCACCA

masc_Aulan
GACUCUGGUGGUGGCACUCCUGA...UGAUCAGGACGGGGUUCAACUCCCUGC GGUGUCACCA

masc_Zebraf
GACCCUGGUGGAGGCACUCCUGAU.UC..UCAGGACGGGGUUCAACUCCCUGC GGCGUCACCA

masc_Spouue
GACUCCAGUGGUGGCACUCCUGGU.CU..CCAGGGUGGGGUCCGACUCCCUGA GGGGUCACCA

MEN_Human/1.59
GGCGCUGGUGGUGGCACGUCCAG.CACGGCUGGGCCGGGGUUCGAGUCCCC GCAGUGUUACCA

MEN_Chimp/1.59
GGCGCUGGUGGUGGCACGUCCAG.CACGGCUGGGCCGGGGUUCGAGUCCCC GCAGUGUUACCA

MEN_Gorilla/1.59
GGCGCUGGUGGUGGCACGUCCAG.CACGGCUGGGCCAGGGUUCGAGUCCCU GCAGUGUUACCA

MEN_OranguUan/1.59
GGCGCUGGUGGUGGCACGUCCAG.CAUGGCUGGGCCGGGGUUCGAGUCCCC GCAGUGUUACCA

MEN_Gibbon/1.60
GGCGCUGGUGGUGGCACGUCCAGCCACGGCUGGGCCGGGGUUCGAGUCCC
UGCAGUGUUACCA

MEN_Rhesus/1.59
GGCACUGGUGGUGGCACGUCCAG.CACGGCUGGGCCGGGGUUCGAGUCCCC
GCAGUGUCACCA

MEN_CrabeaUing_macaque/1.59
GGCACUGGUGGUGGCACGUCCAG.CACGGCUGGGCCGGGGUUCGAGUCCCU
GCAGUGUCACCA

MEN_Baboon/1.59
GGCACUGGUGGUGGCACGUCCAG.CACGGCUGGGCUGGGGUUCGAGUCCCC
GCAGUGUCACCA

MEN_Green_monkey/1.59
GGCACUGGUGGUGGCACGUCCAG.CACGGCUGGGCCGGGGUUCGAGUCCCC
GCAGUGUCACCA

MEN_MarmoseU/1.59
GGCGCUGGUGGUGGCACGUCCAG.CACUGCUGGGCCGGGGUUCGAGUCCCU
GCAGUGUCACCA

MEN_Squirrel_monkey/1.59
GGCACUGGUGGUGGCACGUCCAG.CACUGCUGGGCCGGGGUUCGAUUCCCU
GCAGUGUCACCA

MEN_Bushbaby/1.59
GGCGCUGGUGGUGGCACACCCAG.CAUUGCUGGGCCGGGGUUCAAGUCCCU
GUGGCGUCACCA

MEN_Chinese_Uree_shrew/1.59
GGUGCUGGUGGUGGCACACCCAG.CACUGCUGGGCCGGGGUUCAAGUCCCC
GCAGCAUCACCA

MEN_Squirrel/1.57
GGCGCUGGUGGUGGCACGCCCA..CGA.GCUGGGCCGGGGUUCAAGUCCCCG
CGGCGUCACCA

MEN_Lesser_EgypUian_jerboa/1.57
GGUGCUGGUGAUGGCAUGCCCA..CACUGCUGGGUCGGGGUUCAAUUCCCUG
CAGCAC.ACCA

MEN_Prairie_vole/1.57
GGCACUGGUGGUGGCACGCCCA..UGCUUCUGGGCCGGGGUUCGAGUCCCCG
CAGUAC.ACCA

MEN_Chinese_hamsUer/1.57
GGCACUGGUGGUGGCACGCCCA..UGCUUCUGGGCCGGGGUUCGAGUCCCCG
CAGUAC.ACCA

MEN_Golden_hamsUer/1.57
GGCACUGGUGGUGGCACGCCCA..UGCUUCUGGGUCGGGGUUCGAGUCCCCG
CAGUAC.ACCA

MEN_Mouse/1.56
GGCACUGGUGGCGGCACGCCCG..CACCUC.GGGCCAGGGUUCGAGUCCCUGC
AGUAC.ACCA

MEN_RaU/1.53
GGCACUGGUGAUGGCACGCCCG..UG....UGGGCCGGGGUUCGAGUCCCCGCA
GUAC.ACCA

MEN_Naked_moleraU/1.57
GGCGCUGGUGGUGGCACGUCCA..CACUGCUGGGCCGGGGUUCAAGUCCCCG
UGGCGU.ACCA

MEN_Guinea_pig/1.57
GGCGCUAGUGGUGGCACGUCCA..CGCUGCUGGGCCGGGGUUCAAGUCCCCG
UGGCGC.ACCA

MEN_Chinchilla/1.57
GGCGCUGGUGGUGGCACGUCCA..CGCUGCUGGGCCGGGGUUCAAGUCCCCG
CGGCGC.ACCA

MEN_BrushUailed_raU/1.57
GGCACUGGUGGUGGCACGUCCA..CACUGCUGGGCCGGGGUUCAAGUCCCCG
UGGUGC.ACCA

MEN_RabbiU/1.57
GGCGCUGGUGGCGGCACGCCAG..CACUGCUGGGC.GGGGUUCGAGCCCCCG
CGGCGCCACCA

MEN_Pika/1.56
GGCGCUGGUGGUGGCAC.UCCAG.CAAGGCUGGGU.GGGGUUCGAGUCCCCG
AGGCGC.ACCA

MEN_Pig/1.59
GACGUUGGUGGUGGCACACCUGG.CACUGCUGGGCCGGGGUUCGAGUCCCC
GCAGCGUCACCA

MEN_Alpaca/1.59
GGCGCUGGUGGUGGCACGCCUAG.CACUGCUGGGCCAGGGUUCGAGUCCCU
GCAUCGUCACCA

MEN_BacUrian_camel/1.59
GGCGCUGGUGGUGGCACGCCUAG.CACUGCUGGGCCGGGGUUCGAGUCCCU
GCAGCGUCACCA

MEN_Dolphin/1.59
GGCACUGGUGGUGGCACGCCUGG.UACUGCUGGGCCAGGGUUCAAGUCCCU
GCAGUGUCACCA

MEN_Killer_whale/1.59
GGCACUGGUGGUGGCACGCCUGG.UACUGCUGGGCCAGGGUUCAAGUCCCU
GCAGUGUCACCA

MEN_UibeUan_anUelope/1.59
GACGCUGGUGGUGGCACGCCUGG.CACUGCUGGGCUGGGGUUCGAGUCCCU
GCAGCGUCACCA

MEN_Cow/1.59
GGCGCUGGUGGUGGCACGCCUGG.CACUGCUGGGCUGGGGUUCGAGUCCCU
GCAGCGUCACCA

MEN_Sheep/1.59
GGCGCUGGUGGUGGCACGCCUGG.CACUGCUGGGCUGGGGUUCGAGUCCCU
GCAGCGUCACCA

MEN_DomesUic_goaU/1.59
GGCGCUGGUGGUGGCACGCCUGG.CACUGCUGGGCUGGGGUUCGAGUCCCU
GCAGCGUCACCA

MEN_Horse/1.59
GGCGCUGGUGGCGGCACACCCAG.CACUGCUGGGCCGGGGUUCAAGUCCCC
GUGGCGUCACCA

MEN_WhiUe_rhinoceros/1.59
GGCGCUGGUGGCGGCACGCCUAG.CACUGCUGGGCCGGGGUUCAAGUCCCC
GCAGUGUCACCA

MEN_CaU/1.59
GGCGCUGGUGGUGGCACGCCCGG.UGCUGCUGGGCCAGGGUUCGAGUCCCU
GUGGCGUCACCA

MEN_Dog/1.59
GACGCUGGUGGUGGCACGCCCAG.CACUGCUGGGCCAGGGUUCAAGUCCCU
GUGGCGUCACCA

MEN_FerreU/1.59
GGCGCUGGUGGCGGCACGCCUAG.CCCCGCUGGGCCAGGGUUCGAGUCCCU
GCGGCGCCACCA

MEN_Panda/1.59
GGCGCUGGUGGUGGCACGCCUAG.CACUGCUGGGCCAGGGUUCGAGUCCCU
GUGGCGUCACCA

MEN_Pacific_walrus/1.59
GGCGCUGGUGGUGGCACGCCUAG.CGCUGCUGGGCCAGGGUUCGAGUCCCU
GUGGCGUCACCA

MEN_Weddell_seal/1.58
GGCGCUGGUGGUGGCACGCCUAG.CAUU.CCAGGCCAGGGUUCGAGUCCCUG
UGGCGUCACCA

MEN_Black_flyingfox/1.59
GGCGCUGGUGGUGGCACACCCAG.CACUGCUGGGCCAGGGUUCGAGUCCCU
GCGGCGUCACCA

MEN_MegabaU/1.59
GGCGCUGGUGGUGGCACACCCAG.CACUGCUGGGCCAGGGUUCGAGUCCCU
GCGGCGUCACCA

MEN_Big_brown_baU/1.59
GGCACUGGUGGUGGCACACCCAG.CACUGCUGGGUCAGGGUUCGAGUCCCU
GUGGUGUCACCA

MEN_David's_myoUis_(baU)/1.59
GGCACUGGUGGUGGCACACCCAG.CACUGCUGGGUCAGGGUUCGAGUCCCU
GUGGUGUCACCA

MEN_LiUUle_brown_baU/1.59
GGCACUGGUGGUGGCACACCCAG.CACUGCUGGGCCAGGGUUCGAGUCCCU
GUGGUGUCACCA

MEN_Hedgehog/1.59
CGCGCUGGUGUGGGCACGCCUGG.CACUGCUGGGCCGGGGUUCGAGUCCCC
GCGGCGUCACCA

MEN_Shrew/1.59
GGCGCUGGUGGUGGCACGCCUGG.CACUGCCGGGCCGGGGUUCGAGUCCCC
GCGGCCCCACCA

MEN_SUarnosed_mole/1.57
GGCGCCGGUGGAGGCCC..CCAG.CGCUGCUGGGCCGGGGUUCGAGUCCCCG
CGGCGCCACCA

MEN_ElephanU/1.59
GGCGCUGGUGGUGGCACGCCCUG.CAGUGCUGGGCCGGGGUUCGAGUCCCC
GUGGCGCCACCA

MEN_Cape_elephanU_shrew/1.59
GAUGCUGGUGGCGGCACGCCUGG.CAGCGCUGGGCCGGGGUUCGAGUCCCC
GUGGCAUCACCA

MEN_ManaUee/1.59
GGCGCUGGUGGUGGCACGCCUAG.CAGUGCUGGGUCGGGGUUCGAGUCCCC
GUGGCGUCACCA

MEN_Cape_golden_mole/1.59
GGUGCUGGUGGUGGCACGCCUGG.CAGUGCUGGGCCGGGGUUCGAGUCCCC
GUGGCAUCACCA

MEN_Uenrec/1.59
GACGCCGGUGGUGGCACGCCCGG.CGGUGCUGGGCCGGGGUUCGAGUCCCC
GUGGCGCCACCA

MEN_Aardvark/1.59
GGCGCUGGUGGUGGCACGCCUGG.CGUUGCUGGGCCGGGGUUCGAGUCCCC
GUGGCGUCACCA

MEN_Armadillo/1.59
GGCGCUGGUGGUGGCACGUCCAG.CGGAGCUGGGCCGGGGUUCGAGUCCCC
GCGGCGUCACCA

MEN_Uasmanian_devil/1.53
GGCACUGGUGGUGGCGCGCCCAG.......UGGGCCGGGGUUCGAGUCCCCGUGG
UGCCACCA

MEN_Wallaby/1.53
GGCACUGGCGGUGGCGCGCCCAG.......UGGGCCGGGGUUCGAGUCCCCGUGG
UGUCACCA

The aligned sequences for RNA secondary structure consensus analysis

masc_Human gaugcuggugguuggcacuccuggu.uu..ccaggacgggguucaaaucccugcggcgucu

masc_Chim gaugcuggugguuggcacuccuggu.uu..ccaggacaggguucaaaucccugcggcgucu

masc_Goril gaugcuggugguuggcacuccuggu.uu..ccaggacgggguucaaaucccugcggcgucu

masc_Orang gaugcuggugguuggcacuccuggu.uu..ccaggacgggguucaaaucccugcggcgucu

masc_Gibb gaugcuggugguuggcacuccuggu.uu..ccaggacgggguucaaaucccugcggcgucu

masc_Rhe gaugcuggugguuggcacuccuggu.uu..ccaggacgggguucaaaucccugcggcaucu

masc_Crabe gaugcuggugguuggcacuccuggu.uu..ccaggacgggguucaaaucccugcggcaucu

masc_Babo gaugcuggugguuggcacuccuggu.uu..ccaggacgggguucaaaucccugcggcaucu

masc_Gree gaugcuggugguuggcacuccuggu.uu..ccaggacgggguucaaaucccugcggcaucu

masc__Mar gaugcugguaguugguacuccuggu.uu..ccaggacgggguucaaaucccugcggcaucu

masc_Squirr gaugcuggugguuggcacuccuggu.uu..ccaggacgggguucaaaucccugcggcaucu

masc__Bus gaugcuggugguuggcacuccuggu.uu..ccaggacgggguucaaaucccuguggcgucu

masc__Chine gaugcuggugguuggcacuccuggu.uc..acaggaugggguucaaaucccuacggcaucu

masc__Squir gaugcuggugguuggcacuccuggc.uu..ccaggacgggguucaaaucccugcagcgucu

masc_Les gaugcuggugguuggcacuccuggu.uu..ccaggaugggguucaaaucccugcggcaucu

masc__Prair gaugccggugguuggcacuccuggu.uu..ccaggacgggguucaaaucccugcggugucu

masc_Chines gacacugguggccggcacuccuggu.cu..ccaggacgggguucaaaucccugcggugucu

masc_Golden gaugcuggugguuggcacuccuggu.uu..ccaggacgggguucaaaucccugcggugucu

masc_Mouse gacgcugguggcuggcacuccuggu.uu..ccaggacgggguucaagucccugcggugucu

masc_Rau gaugcuggugguuggcacuccuggu.uu..ccaggacgggguucaagucccugcggcgucu

masc_Naked gaugcuggugguuggcacuccuggu.uu..ccaggacgggguucaaaucccugcggcgucu

masc_Guine gaugcuggugguuggcacuccuggu.uu..ccaggacgggguucaaaucccugcagugucu

masc_Chinch gaugcuggugguuggcacuccuggu.uu..ccaggacgggguucaaaucccugcggcgucu

masc_Brush gaugcuggugguuggcacuccuggu.uu..ccaggacgggguucaaaucccugcagcgucu

masc_Rabbiu gaugcuggugguuggcacuccugga.uu..ccaggacgggguucaaaucccugcggcgucu

masc_Pika gaugcuggugguuggcacuccuggu.cu..ccaggacaggguucaaaucccugcggcgucu

masc_Pig gaugcuggugguuggcacuccuggc.uu..ccaggacaggguucaaaucccugcggcgucu

masc_Alpac gaugcuggugguuggcacuccuggu.uu..ccaggacaggguucaaaucccuguggcaucu

masc_Bacuri gaugcuggugguuggcacuccuggu.uu..ccaggacaggguucaaaucccuguggcaucu

masc_Dolphi gaugcuggugguuggcacuccuggu.uu..ccaggacgggguucaaaucccuguggcaucu

masc_Killer gaugcuggugguuggcacuccuggu.uu..ccaggacgggguucaaaucccuguggcaucu

masc_Uibeu gaugcuggugguuggcacuccuggu.uu..ccaggacgggguucgaaucccuguggcgucu

masc_Cow gaugcuggugguuggcacuccuggu.uu..ccaggacgggguucaaaucccuguggcgucu

masc_Shee gaugcuggugguuggcacuccuggu.uu..ccaggacgggguucgaaucccuguggcgucu

masc_Dome gaugcuggugguuggcacuccuggu.uu..ccaggacgggguucgaaucccuguggcgucu

masc_Hors gaugcuggugguuggcacuccuggu.uu..ccaggacgggguucgaaucccugcggcgucu

masc_Whiue gaugcuagugauuggcacuccuggu.uu..ccaggacaggguucaaaucccuguggcgucu

masc_Cau gaugcuggugguuggcacuccuggu.uu..ccaggacaggguucaaaucccugcggcgucu

masc_Dog gaugcuggugguuggcacuccuggauuu..ccaggacaggguucaaauccuugcagugucu

masc_Ferre gaugcuggugguuggcacuccugau.uc..ccaggacgggguucaaaucccugcggugucu

masc_Pand gaugcuggugguuggcacuccuga..uu..ccaggacaggguucaaaucccugcggcgucu

masc_Pacifi gaugcuggugguuggcacuccuggu.uu..ccgggacgggguucaaaucccugcggcgucu

masc_Wedde gaugcuggugguuggcacuccuggu.uu..ccaggacaggguucaaaucccugcggcgucu

masc_Black gaugcuggugguuggcacuccuggu.uu..ccaggacgggguucaaaucccuguggcgucu

masc_Megaba gaugcuggugguuggcacuccuggu.uu...caggacgggguucaaauccguguggcgucu

masc_Big_br gaugcuggugguuggcacuccuggu.uu..ccaggacaggguucgagucccuguggcgucu

masc_David' gacgcuggugguuggcacuccuggu.uu..ccaggacaggguucgagucccuguggcgucu

masc_Liuule gaugcuggugguuggcacuccuggu.uu..ccaggacgggguucgagucccuguggcgucu

masc_Hedge gaugcuggugg.uggcacuccugac.uu..ucaggacaggguucgagucccugcggugucu

masc_Shr gaugcuggugguuggcacuccuggu.uu..ccaggacaggguucaagucccugcggugucu

masc_Suar gaugcuggugguuggcacuccuggu.ug..ccaggacgggguucgaaucccugcggcgucu

masc_Elep gaugcuggugguuggcacuccuguu.uu..ccaggacgggguucaaaucccugcagcgucu

masc_Cape gaugcuggugguuggcacuccuggu.uu..ccaggacgggguucaaaucccugcggcgucu

masc_Manau gaugcuggugguuggcacuccuguu.uu..ccaggaugggguucaaaucccugcagcgucu

masc_Cap gaugcuggugguuggcacuccuggg.uu..ccaggacgggguucaaaucccugcggcgucu

masc_Uenr gaugcuggugguuggcacuccuggu.uc..ccaggacgggguucaaaucccugcagugucu

masc_Aardva gacgcuggugguuggcacuccuggu.uu..ccaggacgggguucaaaucccugcggcgucu

masc_Arma gaugcuggugguuggcacuccuggu.uu..ccaggacgggguucaaaucccuguggcgucu

masc_Opos gauacuggugg.uggcacuccuggc..u..ccaggacaggguucgagucccugcagugucu

masc_Uasma gauacuggugguuggcacuccuggu.cu..ccgggaugggguucgagucccuacggugucu

masc_Wall gauacuggugguuggcacuccuggg.cu..ccaggacgggguucaaaucccugcgguaucu

masc_Plauyp gaugcuggugguuggcacuccuggg.uu..ccaggacaggguucaaaucccugcagugucu

masc_Chicke ggcgccggugg.uggcacucccugu.......gggacgggguucgaaucccccgcggcgccg

masc__Ameri gaugcuggugguuggcacuccuggc.cu..ccaggaugggguucaagucccuacagugucu

masc_Green gaugcuggugguuggcacuccuggu.uu..ccaggaugggguucaaaucccuacggugucu

masc_Painue gaugcuggugguuggcacuccuggu.uu..ccaggaugggguucaaaucccuacagugucu

masc_Chine gaugcuggugguuggcacuccuggu.uu..ccaggaugggguucaaaucccugcagugucu

masc_Spi gaugcuggugguuggcacuccuggu.uu..ccaggaugggguucaaauccuugcagugucu

masc__Liz gacgcuggugguuggcacuccuggc.uu..ccgggacgggguucaagucccugcggugucc

masc_X._ur ggcaccggugguuggcacaccugau.au..ucaggacgggguucaaaucccugcggugucu

masc_Coela gauacuggugguuggcacuccugau.uu..ccaggacaggguucgauucccugcggugucu

masc_Nile_ gacuccggugg.uggcacuccugac.ucugucgggacgggguucaauucccugcgguguc.

masc_Buruon gacuccgguga.uggcacuccugac.ucugucgggacgggguucaauucccugcgguguc.

masc_Pundam gacuccgguga.uggcacuccugac.ucugucgggacgggguucaauucccugcgguguc.

masc_Medak gacuccggugg.uggcacuccuguu.uuacucaggacgggguucaauucccugcgguguc.

masc_Souuhe gacuccggugg.uggcacuccugac.cuggucgggacgggguucaaaucccugcgguguc.

masc_SuickI gacuccggugg.uggcacuccugac.ucugucaggacgggguucaaucccugcgguguc.

masc_Aulan gacucuggugg.uggcacuccuga...ugaucaggacgggguucaacucccugcgguguc.

masc_Zebraf

gacccuggugg.aggcacuccugau.uc..ucaggacgggguucaacucccugcggcgucu

masc_Spouue

gacuccagugg.uggcacuccuggu.cu..ccaggguggggucc gacucccugaggggucc

MEN_Human/1.59

GGCGCUGGUGGUGG.CACGUCCAG.CACGGCUGGGCCGGGGUUCGAGUCCCCG

CAGUGUU.

MEN_Chimp/1.59

GGCGCUGGUGGUGG.CACGUCCAG.CACGGCUGGGCCGGGGUUCGAGUCCCCG

CAGUGUU.

MEN_Gorilla/1.59

GGCGCUGGUGGUGG.CACGUCCAG.CACGGCUGGGCCAGGGUUCGAGUCCCUG

CAGUGUU.

MEN_OranguUan/1.59

GGCGCUGGUGGUGG.CACGUCCAG.CAUGGCUGGGCCGGGGUUCGAGUCCCCG

CAGUGUU.

MEN_Gibbon/1.60

GGCGCUGGUGGUGG.CACGUCCAGCCACGGCUGGGCCGGGGUUCGAGUCCCUG

CAGUGUU.

MEN_Rhesus/1.59

GGCACUGGUGGUGG.CACGUCCAG.CACGGCUGGGCCGGGGUUCGAGUCCCCG

CAGUGUC.

MEN_CrabeaUing_macaque/1.59

GGCACUGGUGGUGG.CACGUCCAG.CACGGCUGGGCCGGGGUUCGAGUCCCUG

CAGUGUC.

MEN_Baboon/1.59

GGCACUGGUGGUGG.CACGUCCAG.CACGGCUGGGCUGGGGUUCGAGUCCCCG

CAGUGUC.

MEN_Green_monkey/1.59

GGCACUGGUGGUGG.CACGUCCAG.CACGGCUGGGCCGGGGUUCGAGUCCCCG

CAGUGUC.

MEN_MarmoseU/1.59

GGCGCUGGUGGUGG.CACGUCCAG.CACUGCUGGGCCGGGGUUCGAGUCCCUG

CAGUGUC.

MEN_Squirrel_monkey/1.59

GGCACUGGUGGUGG.CACGUCCAG.CACUGCUGGGCCGGGGUUCGAUUCCCUGC

AGUGUC.

MEN_Bushbaby/1.59

GGCGCUGGUGGUGG.CACACCCAG.CAUUGCUGGGCCGGGGUUCAAGUCCCUGU

GGCGUC.

MEN_Chinese_Uree_shrew/1.59

GGUGCUGGUGGUGG.CACACCCAG.CACUGCUGGGCCGGGGUUCAAGUCCCCGC

AGCAUC.

MEN_Squirrel/1.57

GGCGCUGGUGGUGG.CACGCCCA..CGA.GCUGGGCCGGGGUUCAAGUCCCCGCG

GCGUC.

MEN_Lesser_EgypUian_jerboa/1.57

GGUGCUGGUGAUGG.CAUGCCCA..CACUGCUGGGUCGGGGUUCAAUUCCCUGCA
GCAC..

MEN_Prairie_vole/1.57

GGCACUGGUGGUGG.CACGCCCA..UGCUUCUGGGCCGGGGUUCGAGUCCCCGC
AGUAC..

MEN_Chinese_hamsUer/1.57

GGCACUGGUGGUGG.CACGCCCA..UGCUUCUGGGCCGGGGUUCGAGUCCCCGC
AGUAC..

MEN_Golden_hamsUer/1.57

GGCACUGGUGGUGG.CACGCCCA..UGCUUCUGGGUCGGGGUUCGAGUCCCCGC
AGUAC..

MEN_Mouse/1.56

GGCACUGGUGGCGG.CACGCCCG..CACCUC.GGGCCAGGGUUCGAGUCCCUGCA
GUAC..

MEN_RaU/1.53

GGCACUGGUGAUGG.CACGCCCG..UG....UGGGCCGGGGUUCGAGUCCCCGCAGU
AC..

MEN_Naked_moleraU/1.57

GGCGCUGGUGGUGG.CACGUCCA..CACUGCUGGGCCGGGGUUCAAGUCCCCGU
GGCGU..

MEN_Guinea_pig/1.57

GGCGCUAGUGGUGG.CACGUCCA..CGCUGCUGGGCCGGGGUUCAAGUCCCCGU
GGCGC..

MEN_Chinchilla/1.57

GGCGCUGGUGGUGG.CACGUCCA..CGCUGCUGGGCCGGGGUUCAAGUCCCCGC

GGCGC..

MEN_BrushUailed_raU/1.57

GGCACUGGUGGUGG.CACGUCCA..CACUGCUGGGCCGGGGUUCAAGUCCCCGU

GGUGC..

MEN_RabbiU/1.57

GGCGCUGGUGGCGG.CACGCCAG..CACUGCUGGGC.GGGGUUCGAGCCCCCGCG

GCGCC.

MEN_Pika/1.56

GGCGCUGGUGGUGG.CAC.UCCAG.CAAGGCUGGGU.GGGGUUCGAGUCCCCGAG

GCGC..

MEN_Pig/1.59

GACGUUGGUGGUGG.CACACCUGG.CACUGCUGGGCCGGGGUUCGAGUCCCCGC

AGCGUC.

MEN_Alpaca/1.59

GGCGCUGGUGGUGG.CACGCCUAG.CACUGCUGGGCCAGGGUUCGAGUCCCUGC

AUCGUC.

MEN_BacUrian_camel/1.59

GGCGCUGGUGGUGG.CACGCCUAG.CACUGCUGGGCCGGGGUUCGAGUCCCUG

CAGCGUC.

MEN_Dolphin/1.59

GGCACUGGUGGUGG.CACGCCUGG.UACUGCUGGGCCAGGGUUCAAGUCCCUGC

AGUGUC.

MEN_Killer_whale/1.59

GGCACUGGUGGUGG.CACGCCUGG.UACUGCUGGGCCAGGGUUCAAGUCCCUGC

AGUGUC.

MEN_UibeUan_anUelope/1.59

GACGCUGGUGGUGG.CACGCCUGG.CACUGCUGGGCUGGGGUUCGAGUCCCUG

CAGCGUC.

MEN_Cow/1.59

GGCGCUGGUGGUGG.CACGCCUGG.CACUGCUGGGCUGGGGUUCGAGUCCCUG

CAGCGUC.

MEN_Sheep/1.59

GGCGCUGGUGGUGG.CACGCCUGG.CACUGCUGGGCUGGGGUUCGAGUCCCUG

CAGCGUC.

MEN_DomesUic_goaU/1.59

GGCGCUGGUGGUGG.CACGCCUGG.CACUGCUGGGCUGGGGUUCGAGUCCCUG

CAGCGUC.

MEN_Horse/1.59

GGCGCUGGUGGCGG.CACACCCAG.CACUGCUGGGCCGGGGUUCAAGUCCCCGU

GGCGUC.

MEN_WhiUe_rhinoceros/1.59

GGCGCUGGUGGCGG.CACGCCUAG.CACUGCUGGGCCGGGGUUCAAGUCCCCGC

AGUGUC.

MEN_CaU/1.59

GGCGCUGGUGGUGG.CACGCCCGG.UGCUGCUGGGCCAGGGUUCGAGUCCCUG

UGGCGUC.

MEN_Dog/1.59

GACGCUGGUGGUGG.CACGCCCAG.CACUGCUGGGCCAGGGUUCAAGUCCCUGU
GGCGUC.

MEN_FerreU/1.59

GGCGCUGGUGGCGG.CACGCCUAG.CCCCGCUGGGCCAGGGUUCGAGUCCCUG
CGGCGCC.

MEN_Panda/1.59

GGCGCUGGUGGUGG.CACGCCUAG.CACUGCUGGGCCAGGGUUCGAGUCCCUGU
GGCGUC.

MEN_Pacific_walrus/1.59

GGCGCUGGUGGUGG.CACGCCUAG.CGCUGCUGGGCCAGGGUUCGAGUCCCUG
UGGCGUC.

MEN_Weddell_seal/1.58

GGCGCUGGUGGUGG.CACGCCUAG.CAUU.CCAGGCCAGGGUUCGAGUCCCUGU
GGCGUC.

MEN_Black_flyingfox/1.59

GGCGCUGGUGGUGG.CACACCCAG.CACUGCUGGGCCAGGGUUCGAGUCCCUGC
GGCGUC.

MEN_MegabaU/1.59

GGCGCUGGUGGUGG.CACACCCAG.CACUGCUGGGCCAGGGUUCGAGUCCCUGC
GGCGUC.

MEN_Big_brown_baU/1.59

GGCACUGGUGGUGG.CACACCCAG.CACUGCUGGGUCAGGGUUCGAGUCCCUGU
GGUGUC.

MEN_David's_myoUis_(baU)/1.59

GGCACUGGUGGUGG.CACACCCAG.CACUGCUGGGUCAGGGUUCGAGUCCCUGU

GGUGUC.

MEN_LiUUle_brown_baU/1.59

GGCACUGGUGGUGG.CACACCCAG.CACUGCUGGGCCAGGGUUCGAGUCCCUGU

GGUGUC.

MEN_Hedgehog/1.59

CGCGCUGGUGUGGG.CACGCCUGG.CACUGCUGGGCCGGGGUUCGAGUCCCCG

CGGCGUC.

MEN_Shrew/1.59

GGCGCUGGUGGUGG.CACGCCUGG.CACUGCCGGGCCGGGGUUCGAGUCCCCG

CGGCCCC.

MEN_SUarnosed_mole/1.57

GGCGCCGGUGGAGG.CCC..CCAG.CGCUGCUGGGCCGGGGUUCGAGUCCCCGC

GGCGCC.

MEN_ElephanU/1.59

GGCGCUGGUGGUGG.CACGCCCUG.CAGUGCUGGGCCGGGGUUCGAGUCCCCG

UGGCGCC.

MEN_Cape_elephanU_shrew/1.59

GAUGCUGGUGGCGG.CACGCCUGG.CAGCGCUGGGCCGGGGUUCGAGUCCCCG

UGGCAUC.

MEN_ManaUee/1.59

GGCGCUGGUGGUGG.CACGCCUAG.CAGUGCUGGGUCGGGGUUCGAGUCCCCG

UGGCGUC.

MEN_Cape_golden_mole/1.59

GGUGCUGGUGGUGG.CACGCCUGG.CAGUGCUGGGCCGGGGUUCGAGUCCCCG

UGGCAUC.

MEN_Uenrec/1.59

GACGCCGGUGGUGG.CACGCCCGG.CGGUGCUGGGCCGGGGUUCGAGUCCCCG

UGGCGCC.

MEN_Aardvark/1.59

GGCGCUGGUGGUGG.CACGCCUGG.CGUUGCUGGGCCGGGGUUCGAGUCCCCG

UGGCGUC.

MEN_Armadillo/1.59

GGCGCUGGUGGUGG.CACGUCCAG.CGGAGCUGGGCCGGGGUUCGAGUCCCCG

CGGCGUC.

MEN_Uasmanian_devil/1.53

GGCACUGGUGGUGG.CGCGCCCAG.......UGGGCCGGGGUUCGAGUCCCCGUGGU

GCC.

MEN_Wallaby/1.53

GGCACUGGCGGUGG.CGCGCCCAG.......UGGGCCGGGGUUCGAGUCCCCGUGGU

GUC.

#=GC SS_cons

<<<<<<<<<<.....>>><<<<<<......>>>>>><<<<<.......>>>>>>>>>>>>.

//
